# The nature of active sites for carbon dioxide electroreduction over oxide-derived copper catalysts

**DOI:** 10.1038/s41467-020-20615-0

**Published:** 2021-01-15

**Authors:** Dongfang Cheng, Zhi-Jian Zhao, Gong Zhang, Piaoping Yang, Lulu Li, Hui Gao, Sihang Liu, Xin Chang, Sai Chen, Tuo Wang, Geoffrey A. Ozin, Zhipan Liu, Jinlong Gong

**Affiliations:** 1 Key Laboratory for Green Chemical Technology of Ministry of Education, School of Chemical Engineering and Technology, Tianjin University, 300072 Tianjin, China; 2 Collaborative Innovation Center of Chemical Science and Engineering (Tianjin), 300072 Tianjin, China; 3 Department of Chemistry, University of Toronto, Toronto, ON Canada; 4 Collaborative Innovation Centre of Chemistry for Energy Material, Shanghai Key Laboratory of Molecular Catalysis and Innovative Materials, Key Laboratory of Computational Physical Science, Department of Chemistry, Fudan University, Shanghai, China; 5 Joint School of National University of Singapore and Tianjin University, International Campus of Tianjin University, Binhai New City, 350207 Fuzhou, China

**Keywords:** Heterogeneous catalysis, Electrocatalysis

## Abstract

The active sites for CO_2_ electroreduction (CO_2_R) to multi-carbon (C_2+_) products over oxide-derived copper (OD-Cu) catalysts are under long-term intense debate. This paper describes the atomic structure motifs for product-specific active sites on OD-Cu catalysts in CO_2_R. Herein, we describe realistic OD-Cu surface models by simulating the oxide-derived process via the molecular dynamic simulation with neural network (NN) potential. After the analysis of over 150 surface sites through NN potential based high-throughput testing, coupled with density functional theory calculations, three square-like sites for C–C coupling are identified. Among them, Σ3 grain boundary like planar-square sites and convex-square sites are responsible for ethylene production while step-square sites, i.e. *n*(111) × (100), favor alcohols generation, due to the geometric effect for stabilizing acetaldehyde intermediates and destabilizing Cu–O interactions, which are quantitatively demonstrated by combined theoretical and experimental results. This finding provides fundamental insights into the origin of activity and selectivity over Cu-based catalysts and illustrates the value of our research framework in identifying active sites for complex heterogeneous catalysts.

## Abstract (web summary)

The active sites over oxide-derived copper (OD-Cu) catalysts for CO_2_ electroreduction are unclear. Here, the authors show atom-level product-specific active sites on OD-Cu surface models, where planar and convex square sites are responsible for ethylene while the step square site favours alcohols generation.

## Introduction

The excessive utilization of fossil fuels has led to dramatic anthropogenic climate change and serious energy crisis. Electrochemical carbon dioxide reduction (CO_2_R) to renewable fuels and feedstocks has gained significant attention, and been regarded as a promising strategy to close the carbon cycle and store renewable energy^1–3^. Copper is by far the most promising metal capable of converting CO_2_ into multi-carbon hydrocarbons and oxygenates^4–8^. Among the Cu-based catalysts, oxide-derived copper (OD-Cu) is particularly attractive catalyst due to its remarkable selectivity towards potentially valuable C_2+_ products at relatively low overpotentials, which exhibits the prospect of large-scale industrialization^9–11^.

The outstanding catalytic performance for C_2+_ products over OD-Cu catalysts is under intense debate, which is possibly related to, for instance, subsurface oxygen^12^, grain boundaries^10,13^, exposed facet^14^, and local pH^15^. Moreover, recent study by Ager et al.^16^ showed that different C_2+_ products are generated from specific active sites on OD-Cu via isotope labeling. However, the current understanding of atom-scale active sites over OD-Cu falls short of expectation, impeding the rational design of catalysts converting CO_2_ to specific product, like ethylene and ethanol, with high selectivity. The difficulty is that available characterization techniques cannot detect the complex surface features of OD-Cu due to the insufficient spatial resolution. Meanwhile, the computational demanding quantum mechanics (QM) level calculations are impractical for large-scale and long-term simulation, thus inhibiting the display of the “oxide-derived” process and further comprehension of atomic structures on authentic OD-Cu surface models. Therefore, identifying atom-scale product-specific active sites for CO_2_R over OD-Cu catalysts remains grand challenges.

The development of machine-learning-based new calculation methods is a frontier in the field of theoretical catalysis, at the same time, a promising route to analysis the active sites in complex heterogeneous catalytic system^17,18^. Goddard and co-workers^19^ computationally synthesized copper nanoparticles (Cu NP) by simulating the chemical vapor deposition (CVD) process and used machine-learning method to explore the active sites for C–C coupling. However, it is unclear that the surface structure of Cu NP can be compared to that of OD-Cu. OD-Cu differs from Cu NP or poly-Cu in that it shows higher selectivity for C_2+_ oxygenates at lower overpotential^9,10^, which means that active sites for C_2+_ products on OD-Cu may be different from that on Cu NP. In addition, active sites for specific products should be further clarified. Thus, new calculation method should be developed to simulate the “oxide-derived” process to obtain the authentic OD-Cu surface model.

Here, we break the spatiotemporal limitation of QM and use molecular dynamic simulation with global neural network potential (NN-MD)^20,21^ to simulate the whole dynamic evolution of the surface from copper oxide to OD-Cu. By combining NN potential-based high-throughout testing, density functional theory calculations and experimental studies, we establish the linkage correlations between the atomic structure of active sites and their catalytic activities for specific products in CO_2_R over OD-Cu catalysts. This work provides theoretical guidance for the design of highly selective Cu-based catalysts and develops a versatile research framework, machine-learning accelerated theoretical calculations, coupled with actionable experiments, for analyzing atomic structures of active sites in heterogeneous catalytic systems.

## Results

**Fig. 1:**
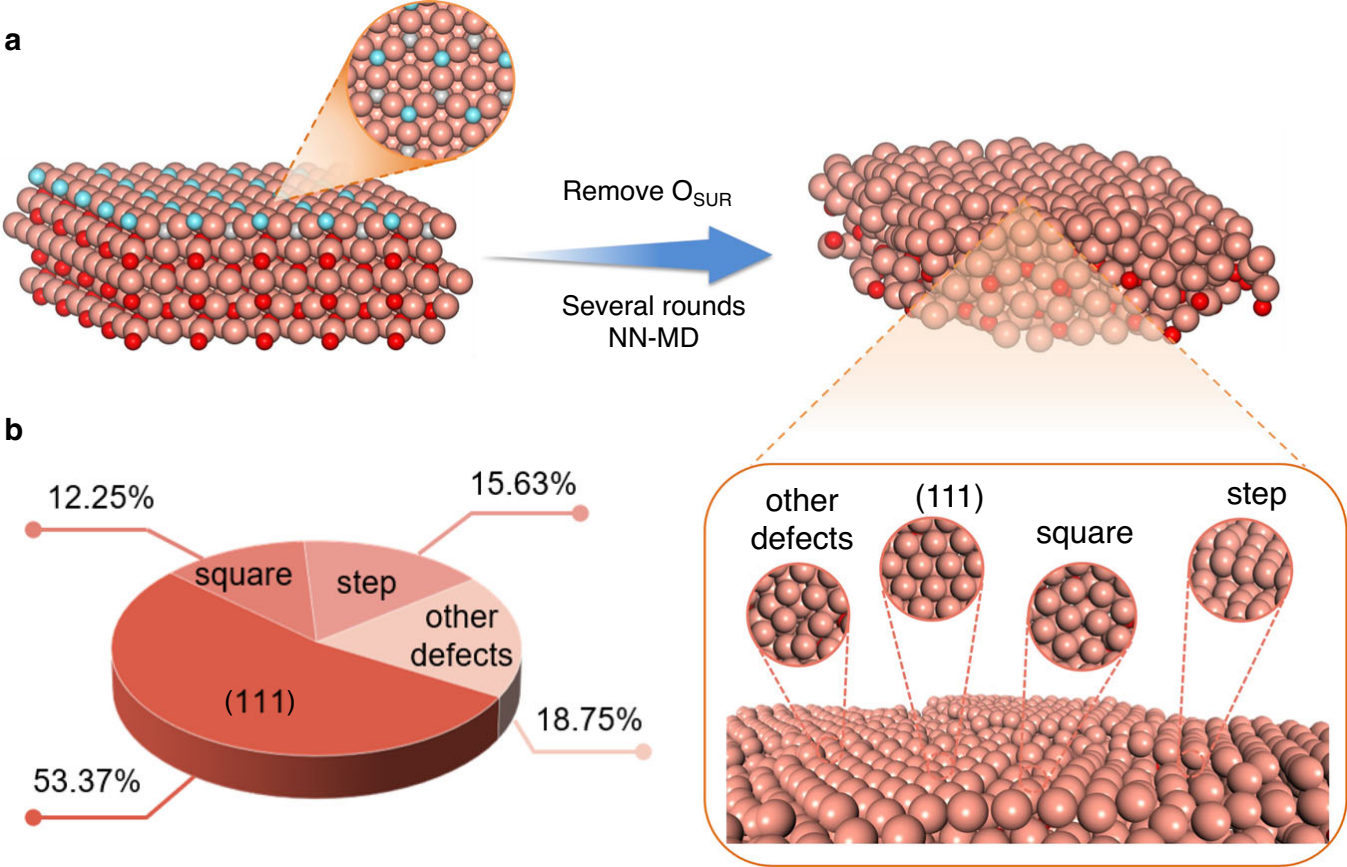
Theoretical simulation of oxide-derived process. **a** Illustration of our procedure to construct OD-Cu model with molecular dynamic simulation. Color code: brown-Cu; blue-surface O; gray-subsurface O; red-bulk O. **b** Proportions of different surface structures.

A realistic surface model is the prerequisite for the exact analysis of atomic structure of active sites. We employed molecular dynamics simulation with neural network potential (NN-MD)^20,21^ to simulate the whole reduction process of the oxide precursor. Experimentally, OD-Cu are mainly derived from Cu_2_O phase^9,22^, thus Cu_2_O (111), the most stable facet^23^, was chosen as the precursor for the reduction process. To simulate the “oxide-derived” process, the two-step approach was employed. First, NN-MD was performed with canonical ensemble (NVT) to obtain a thermodynamically balanced surface structure. Subsequently, the oxygen vacancy formation energies (*E*_ov_) of surface O were calculated, wherein O atoms with lower vacancy formation energies were removed to represent the reduction process, thereby forming a partially reduced surface. Afterwards, another round NN-MD simulation was carried out followed by the reduction, i.e., removal of surface O atoms, until O can no longer migrate to the surface and all been trapped in the third layer or below (Fig. 1a and Supplementary Figs. 1–12). (for details, see the “Methods”) Previous research has shown that O below the third layer had little effect on the adsorption behavior of adsorbates^24^, therefore we believe that our OD-Cu surface models are consistent with the current experimental research that metallic copper is the active phase for CO_2_R over OD-Cu rather than the oxide phase^25^.

In our simulated OD-Cu surface structures, (111) facet dominates, occupying about 53% surface area (Fig. 1b). Besides, there are some obvious steps, which resemble Cu(S)-[*n*(111) × (111)] step sites, where one atomic height of (111) step is introduced into several atomic rows of (111) terrace. Moreover, a few defects like grain boundaries and vacancies exist (Supplementary Fig. 13). Up to now, authentic OD-Cu surface structures are obtained based on NN-MD simulation of the whole reduction process.

**Fig. 2:**
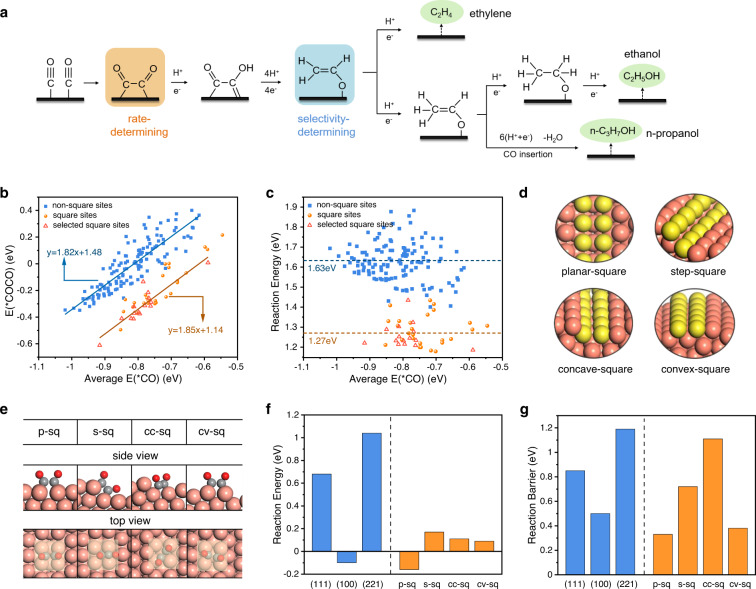
Identification of C–C coupling active sites. **a** Reaction pathway for C_2+_ products in CO2R. **b** The adsorption energy of *COCO as the function of the average adsorption energy of two adsorbed *CO. **c** Reaction energy (2*CO → *COCO) as the function of the average E(*CO) on these sites. **d** DFT periodic slab models of four square-like sites. **e** *OCCO configurations on these four sites (solvent molecules have been removed to show the adsorbate configurations). **f** Reaction energies and **g** barriers (2*CO → *COCO) on different DFT slab models under appropriate electrochemical interface. Color code: brown-Cu; yellow-Cu in square-like sites; gray-C; red-O.

To improve the activity and selectivity for C_2+_ products of the catalyst, it is crucial to understand the nature of the active sites presented on Cu catalyst. The pathway for C_2+_ products^26–28^ is illustrated in Fig. 2a. Solid experimental researches show that CO_2_ electroreduction to multi-carbon products is independent on the standard hydrogen electrode (SHE) scale and C–C coupling step that does not involve proton transfer is considered as rate-determining step (RDS)^29^, which means the rate of C–C coupling determines the whole activity for C_2+_ products over OD-Cu catalysts. Both vibrational spectra^30,31^ and kinetic models^32,33^ reveal that *CO is the main intermediate on Cu surface under reduction conditions. Considering that C_2+_ pathway displays a pH independent on SHE scale, 2*CO dimerization is thus the most accepted form for C–C coupling on copper surface^34^.

We then randomly selected 155 surface sites on OD-Cu surface models to explore this dimerization process. As shown in Fig. 2b, some sites obviously show stronger *COCO binding energies. Interesting, these sites have the same feature that they all own the square-like orientation. We then selected another 15 square sites from OD-Cu surfaces to verify this tendency. Not surprisingly, these square sites can efficiently stabilize the *COCO intermediate as well. Two disparate scaling relationship can be obtained between the average adsorption energy of two binding *CO (*E*(CO)) and coupled intermediate *COCO (*E*(COCO)) on the non-square sites and square sites. Moreover, these two relationships almost share the same slope but have a 0.34 eV intercept difference. This value is very close to the adsorption energy difference, 0.31 eV, of *COCO over Cu(111) and Cu(100), indicating the decreased intercept is merely attributed to the additional stabilizing effect for *COCO intermediate at square-like sites. Reflected in reaction energy of C–C coupling process (Fig. 2c), the average reaction energy on square sites is 1.27 eV, 0.36 eV lower than that on non-square sites. Therefore, square-like sites are the most potential candidates to serve as active sites for C–C coupling.

Further analysis found that these square sites have different local structures. Therefore, we classified these sites into four types: planar-square (p-sq), step-square (s-sq), concave-square (cc-sq) and convex-square (cv-sq). p-sq sites are the Σ3 grain boundary created by two flat (111) planes with 60° lattice orientation difference. s-sq sites are similar to Cu(S)-[*n*(111) × (100)] step sites where (100) steps are introduced to (111) basal plane. While cc-sq and cv-sq are formed by rotation and then splicing of two (111) planes. In order to explore C–C coupling more accurately, we abstracted these four surface sites into small slab models for accurate density functional theory (DFT) calculations (Fig. 2d), which are expected to have similar chemical properties to the selective sites from the MD models (Methods and Supplementary Tables 4–6). Here, (322) and (221) facet, 5(111) × (100) and 5(111) × (111), represent s-sq sites and other step sites, respectively.

Our calculation shows that CO dimerization reaction over these four square-like structures are less endothermic than that over (111) and (221) facet (Fig. 2e, f) under proper electrochemical interface. Among them, p-sq exhibits the lowest reaction energy of −0.16 eV. Moreover, we found that the reaction barriers of C–C coupling on p-sq, s-sq and cv-sq sites are 0.33 eV, 0.72 eV, and 0.39 eV, respectively, much lower than 0.85 eV and 1.19 eV on (111) and (221) facets (Fig. 2g). Based on the analysis above, three active sites, p-sq, s-sq and cv-sq sites, which favor for C_2+_ products production have been identified on OD-Cu.

**Fig. 3:**
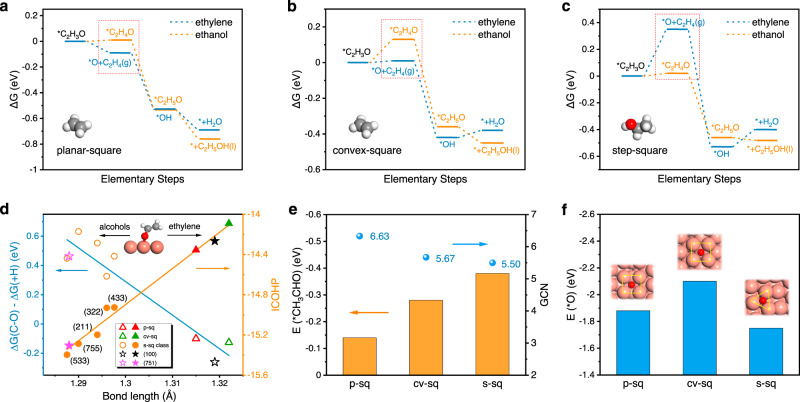
Identification of product-specific active sites. Energy profile to form ethylene (blue line) and ethanol (orange line) for **a** planar-square site, **b** convex-square site, and **c** step-square site. **d** Blue: The relationship between the bond length of C–O in adsorbed CH_2_CHO and the difference of Gibbs-free reaction energy of C–O scission (ΔG(C–O)) and hydrogenation (ΔG(+H)). Orange: The relationship between the bond length of C–O in adsorbed CH_2_CHO and the ICOHP of C–O bond. **e** General coordination number and adsorption energy of *CH_3_CHO on these three active sites. **f** Adsorption energy of *O on these three active sites.

Previous work proved that ethylene and ethanol are not generated from the same types of active sites, which means the C–C coupling active sites can be further subdivided^16^. From calculated free energy profiles of the whole reaction pathway (Supplementary Figs. 24–26), *CH_2_CHO serves as the selectivity determining intermediate (SDI), which corresponds to previous calculations on copper single crystal facets^27,28^. The ethylene pathway proceeds with cleavage of C–O bond while alcohols are formed by further protonation. On p-sq and cv-sq sites (Fig. 3a, b), the cleavage of C–O bond to form C_2_H_4_ is thermodynamic favored compared to the protonation of α-C to form *CH_3_CHO intermediate. Conversely, s-sq site shows strong thermodynamic tendency for further hydrogenation, thus inhibiting the C_2_H_4_ pathway and meanwhile promoting alcohols production (Fig. 3c). These results suggest that p-sq and cv-sq sites are responsible for generating ethylene while s-sq favors the alcohols production.

Interestingly, we found that the tendency to produce ethylene or alcohols can be simply predicted by the C–O bond length of *CH_2_CHO. The shortest C–O bond length (1.296 Å) is in *CH_2_CHO on s-sq site, followed by that on p-sq and cv-sq sites (1.315 Å and 1.322 Å). To further quantitatively evaluate the strength of the bond, we conducted the integrated crystal orbital Hamilton population (ICOHP) analysis of C–O bond (Fig. 3d). The shorter bond length and larger value of –ICOHP indicates that the C–O bond is harder to break, which means the ethylene pathway is inhibited while alcohols pathway is favored. Indeed, the difference between the Gibbs-free reaction energy to break C–O bond and further protonation is negatively correlated to the C–O bond length (Fig. 3d). Remarkably, s-sq class, the Cu(211), Cu(533), Cu(755) and Cu(433) [*n* = 3, 4, 6, 7 in *n*(111) × (100)] all exhibit the same tendency like (322) facet. It is noteworthy, that, Cu(100) and Cu(751), which experimentally show more selective for ethylene and alcohols^7^, fall in their respective product region, thus meaning the bond length of C–O in adsorbed *CH_2_CHO may be a simple descriptor for the selectivity of ethylene and alcohols on different surface structures.

The specific geometry of step-square site explains the difference in selectivity. Figure 3e shows that the general coordination number (GCN) of s-sq is the lowest (5.50), thus showing the strongest adsorption energy of acetaldehyde intermediate (*CH_3_CHO), leading to the hydrogenation pathway dominances. Meanwhile, p-sq and cv-sq can supply the fourfold sites to stabilize the newly formed *O after C–O cleavage. While on s-sq site, *O can only adsorb in adjacent triple site, resulting in the instability of *O, thereby further hindering the ethylene pathway (Fig. 3f). Based on the analysis above, we elucidated how the geometry of C–C active sites affects the bifurcation pathway, resulting in the difference of the selectivity for C_2+_ products. Furthermore, we can propose that the key to generating ethanol is that square-like sites coupled with low coordinated sites, where square-like sites are responsible for C–C coupling, low coordinated sites are conducive to further protonation for alcohols. Very recent study^35^ found that square, four-atom Cu islands are the active sites for generating ethanol. This structure certainly meets our design criteria for alcohols production. Although our OD-Cu models didn’t directly observe such structure due to the scale limitation, their conclusion is consistent with ours.

**Fig. 4:**
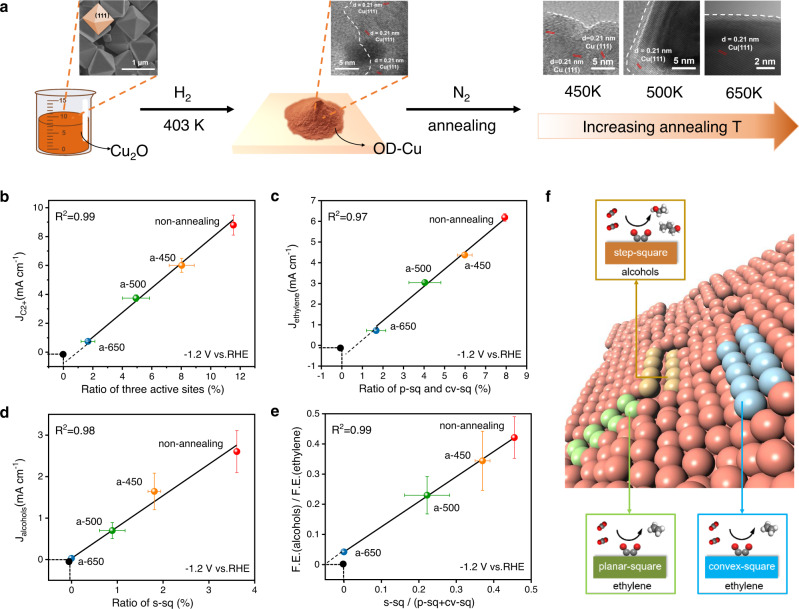
Verification of active sites via thermal annealing process. **a** Schematic illustration of OD-Cu preparation and annealing process in experiment. The upper layer, TEM image of Cu_2_O, SEM images of OD-Cu, a-450, a-500, and a-650 from left to right. The white dotted lines indicate the rough particle surface. The correlation between the current density of **b** C_2+_ products and the ratios of all three active sites **c** ethylene and the ratios of planar-square and convex-square sites **d** alcohols and the ratios of step-square sites. **e** Alcohols/ethylene ratios as the function of the ratios of step-square sites to the sum of planar-square and convex-square sites at −1.2 V vs. RHE. **f** Proposed structure–activity relationship diagram on OD-Cu in CO_2_R. a-450, a-500, and a-650 represent annealing at 450, 500, and 650 K, respectively. The error bars represent the standard deviation from three independent measurements.

To further illustrate the correlation between the catalytic activity for certain C_2+_ products on OD-Cu and these active sites, we experimentally synthesized OD-Cu catalysts, which are derived by Cu_2_O octahedron, where (111) facet dominates. Then, we varied defect concentrations by annealing the OD-Cu at 450, 500, and 650 K (Fig. 4a and Supplementary Figs. 14 and 15). Meanwhile, similar annealing operations were performed theoretically by NN-MD simulation to change the amount of the active sites, thus we can accurately count the ratios of active sites under different annealing temperature conditions from theoretical models (for details, see the “Methods”). After annealing, step sites and some defects are erased in this process and transform into flat (111) facet in general (Supplementary Figs. 16 and 17), which is consistent with previous work that the surface roughness decreased after annealing^10^ and our cyclic voltammetry (CV) curves results (Supplementary Fig. 18).

The ratio of three theoretically identified active sites, p-sq, s-sq, and cv-sq sites, decreased with increasing annealing temperature as well. From our theoretical models, the OD-Cu surface before annealing has the largest proportion, 11.54%, of these three active sites. The percentage drops to 9.37%, 4.93% and 1.67% after annealing at 450, 500, and 650 K, respectively. When plotted against the catalytic activity, a linear relation is obtained between the surface area-normalized current density of C_2+_ products at −1.2 V vs. RHE and the proportion of these three active sites (Fig. 4b). More specifically, the experimental current densities of ethylene and alcohols are linearly correlated to the density of sum of the density of p-sq and cv-sq sites, and the density of s-sq sites, respectively (Fig. 4c, d). The linear correlations are also existed at −1.0 and −1.1 V vs. RHE (Supplementary Figs. 21 and 22). Moreover, the ratio of catalytic activities of alcohols to ethylene is proportional to the ratio of the amount of s-sq to (p-sq + cv-sq) (Fig. 4e). If we change the ordinate from F.E.(alcohols)/F.E.(ethylene) to F.E.(ethylene)/F.E.(alcohols), the positive correlation no long exists (Supplementary Fig. 23), which means the catalytic activities of alcohols and ethylene have no correlation with (p-sq + cv-sq) and s-sq, respectively. This in turn suggests that the CO_2_ reduction activity for ethylene and alcohols strongly depends on their specific active sites. Notably, the intercepts of these relations are all close to 0, meaning that almost all the catalytic activity for these products arises from their specific active sites. Based on this quantitative structure–activity relationship, we can demonstrate that p-sq and cv-sq sites are responsible for ethylene production while s-sq sites promote generating alcohols on OD-Cu surface (Fig. 4f). Therefore, the key to boosting the highly valuable C_2+_ alcohols production is to synthesize Cu-based catalysts with more step-square sites by adjusting the precursors or the synthesis conditions.

Although the activities of ethylene and alcohols both showed a downward trend with the increase of annealing temperature, it can be clearly seen that the activity to produce alcohols decreases faster, resulting in the decreased selectivity of alcohols to ethylene with the increasing annealing temperature (Fig. 4e). The reason is that in the annealing process, the steps are easily erased, leading to the fast decrease of the s-sq coverage. However, the grain boundary like p-sq sites are stuck in two (111) planes, which are relatively robust, leading to the activity of ethylene decreases more slowly.

This work reveals the atomic-level product-specific active sites of CO_2_R over OD-Cu catalysts by combining theoretical and experimental methods, where planar-square and convex-square sites are identified to be the active sites for ethylene production while step-square sites are responsible for the generation of C_2+_ alcohols. These new insights resolved the long-term debate over the active sites on OD-Cu catalysts and identified how each site works, which are the prerequisites for the development of highly selective, i.e., C_2+_ oxygenates, Cu-based catalysts for CO_2_R in the future. Furthermore, this work also provides a versatile tool for the analysis of the complicated heterogeneous catalytic system.

## Methods

### Computational details

All simulations with neural network potential were carried out by LASP software (www.lasphub.com)^20,21^. Molecular dynamic simulation with neural network potential (NN-MD) was performed at a constant temperature (T = 300 K) within the NVT ensemble. Details in neural network potential are seen in Supplementary Methods.

Simulation of the oxide-derived process: We started this process with pristine Cu_2_O (111). Previous studies showed that surface O are unstable on Cu_2_O at −0.1 V (vs. RHE)^36^. Therefore, all the surface O atoms in our slab model, which share the same environment, were removed without distinction. Then, we carried out 1 ns NN-MD at 300 K to obtain the first-stage in the reduction process. After 1 ns simulation, the surface reconstruction was severe and the morphology became chaotic. Some near-surface bulk O migrated to the surface. At this time, the chemical environments of surface O were diverse. It is unreasonable to remove all the surface O at this time simultaneously. Thus, we consider the *E*_ov_ as the criterion to remove surface O. The *E*_ov_ of all the surface oxygen has been calculated by NN potential (The NN potential calculated *E*_ov_ are shown in Supplementary Table 3, leading to RMSE (the root mean squared error) = 0.12 eV). In general, we found that 3-coordinated (3-coor) oxygen correspond to the lower oxygen vacancy formation energy, which is consistent with the instability of 3-coor O on the perfect Cu_2_O surface^23^. Therefore, when removing surface O, coordination number is used as the first criterion and the *E*_ov_ as the second. The one kind is that all 3-coor surface O are removed. The another is that 4-coor O whose *E*_ov_ locates in the range of *E*_ov_ of 3-coordinated O are removed together with 3-coor O. Through this standard, two types of second-stage structures were obtained. Subsequently, 1 ns NN-MD simulation was performed on these two structures to approach equilibrium. Next step for removing O was the same as above until no oxygen can move to the surface. All surface structures are shown in Supplementary Fig. 13, which are marked with OD-Cu-M(M = 1–6). It was worth noting that in the process of NN-MD simulation, some structures have collapsed and cannot maintain the structure of slab owing to the periodicity of slab models, which were not considered in our work.

The calculation of adsorption energy of *CO and *COCO on OD-Cu surface are conducted by neural network potential as well. In order to verify the accuracy of this NN potential, we randomly selected 50 and 15 surface sites on OD-Cu-1 for costly DFT single point calculation for E(*CO) and E(*COCO). The root mean square error (RMSE) of *CO and *COCO adsorption energy between the NN prediction and DFT calculation is as low as 0.08 eV and 0.13 eV, respectively. (Supplementary Tables 4 and 5)

The process of annealing at 400 K: OD-Cu is simulated at 330, 360, 400, and 450 K for 0.2, 0.2, 0.2, and 0.6 ns in order, to simulate the process of heating process, and then simulated at 400, 360, 330, and 300 K for 0.2 ns, respectively, in order, to simulate the process of cooling. The process of annealing at 500 K: OD-Cu is simulated at 360, 420, and 500 K for 0.2, 0.2, and 0.6 ns in order, to simulate the process of heating process, and then simulated at 420, 360, and 300 K for 0.2 ns, respectively in order, to simulate the process of cooling. The process of annealing at 650 K: OD-Cu is simulated at 400, 500, 600, and 650 K in order of 0.2, 0.2, 0.2, and 0.6 ns to simulate the process of heating, and then at 600, 500, 450, and 300 K in order, to simulate the process of cooling at 0.2 ns.

In our article, we performed 0.2 ns NN-MD in each heating and cooling stage. We then changed the residence time (0.1 ns and 0.3 ns) at different annealing temperatures. As a result, we found that the percentages of each structural motifs remain basically unchanged at different annealing temperature (Supplementary Table 10), which means, at least for NN-MD, the annealing time does not change the trend of the ratio of active sites. In all, 0.2 ns is enough for obtaining the ratio of each structural motifs considering the computational cost.

Density functional theory calculations were performed using the plane-wave-based Vienna Ab Initio Simulation package (VASP)^37^. The electron exchange and correlation effects were described by the generalized gradient approximation (GGA) in the form of the Perdew-Burke-Ernzerhof (PBE) functional^38^. The D3 correction method was employed to illustrate the long-range dispersion interactions between the adsorbates, water and Cu surface^39^. The interaction between atomic cores and electrons was described by the projector augmented wave (PAW) method^38^. A cutoff energy of 400 eV for the plane-wave basis set and an atomic force convergence of 0.02 eV/Å were employed. The transition state search was conducted with climbing image nudged elastic band (CI-NEB) method^40^, followed by the dimer method to converge the saddle point within 0.05 eV/Å.

When theoretically investigating electrochemical barriers, it is essential to model suitable electrochemical interface. Previous study has found that one layer of water can accurately describe the adsorption behavior compared to more water layers and a simple hydronium ion can induce the same electric field as cation does^41–43^. Thus, we established one layer of water with an added hydronium ion to simulate the electrochemical interface.

(111), (100), and (221) facets were modeled using (3 × 3), (3 × 3), and (1 × 3) supercells with (3 × 3 × 1) k-point grid. (1 × 3) supercells were used to model step-square sites (*n*(111) × (100)): (211), (533), (322), (755) and (433) facets, (3 × 3 × 1), (3 × 3 × 1), (3 × 3 × 1), (2 × 3 × 1) and (2 × 3 × 1) k-point grid were used. (2 × 1) supercell was used to model (751) facet and (2 × 3 × 1) k-point grid were used. Four layers were used for above models, where the two bottom layers were kept fixed in optimization. Planar-square is the grain boundary created by two flat (111) planes with different lattice orientations. (1 × 3 × 1) k-point grid was used. Concave-square and convex-square are obtained by rotation and then splicing of two (111) planes. (3 × 3 × 1) k-point grid was used for both.

### Materials

KHCO_3_, NaOH, ethanol, and isopropanol were all purchased from Tianjin Kemiou Chemical Reagent Co., Ltd. Polyvinylpyrrolidone (PVP, Mw = 15000, K15) was purchased from Tokyo Chemical Industry Co., Ltd. l-ascorbic acid was purchased from Sinopharm Chemical Reagent Co., Ltd. All reagents were used without any purification process. Ultra purity water (18.25 MΩ cm) supplied by a UP Water Purification System was used in the whole experimental processes. CO_2_, Ar, H_2_, N_2_ all were supplied by Air Liquid (≥99.999%).

### Preparation of Cu_2_O octahedron

The synthesis of Cu_2_O octahedron nanoparticle by a facile method has been described previously^44^. In a typical synthesis, 1.5 g PVP was dissolved into an aqueous solution of 0.01 M CuCl_2_ ∙ 2H_2_O (100 mL) under constant stirring and heated in 55 °C water bath. Then 2.0 M NaOH aqueous solution (10 mL) was added dropwise into the above transparent light green solution. After stirring for 0.5 h, 0.6 M ascorbic acid solution (10 mL) was added dropwise. The color of the solution gradually changed to turbid red. The mixed solution was kept under constant stirring and in 55 °C water bath for 3 h. Then the resulting precipitate was collected by centrifugation and washed with distilled water and ethanol over three times to remove the residual inorganic ions and polymer. Finally, the powder was dried in vacuum at 55 °C for 5 h.

### Preparation of OD-Cu

The prepared Cu_2_O octahedron was placed in a tube furnace (Hefei KeJing Materials Technology Co., LTD) with flowing H_2_ at 200 sccm and heated at 130 °C for 2 h. The reduced sample was cooled slowly to room temperature in H_2_.

### Preparation of a-450, a-500, and a-650

The OD-Cu powder was placed in a tube furnace in 200 sccm flowing N_2_ and heated at 450, 500, and 650 K for 2 h, respectively. The annealed electrodes were cooled slowly to room temperature in N_2_.

### Characterization

Field-emission scanning electron microscopy (FESEM) (Hitachi S-4800, 3 kV) was used to characterize the morphology and microstructure of the samples. Transmission Electron Microscopy (TEM), High-resolution TEM (HRTEM) images were obtained at 200 kV (JEOL JEM-2100F).

### Working electrode preparation

In all, 10 mg of the catalyst was dispersed in 1 mL of isopropanol, including 20 μL of Nafion solution (5 wt%) by sonicating for 1 h. Twenty microliters of the obtained solution was then loaded on a glassy carbon electrode (Gaossunion). The geometric surface area was 0.5027 cm^2^. The electrode was dried in ambient air for the subsequent CO_2_ electroreduction test.

### Electrochemical measurement

Electrochemical tests were performed in a two-compartment H-cell (Supplementary Fig. 27). An anion exchange membrane (FAA-3-PK-130, Fumatech) was used. The electrolyte was 30 mL of 0.1 M KHCO_3_ solution saturated with CO_2_ gas in the cathode part for at least 30 min prior to the CO_2_ reduction test. During electrolysis, CO_2_ was constantly bubbled through the electrolyte at a flow rate of 10 sccm to prevent depletion of CO_2_ in the electrolyte and to allow continuous analysis of gaseous products via a gas chromatograph. The flow rate of CO_2_ was controlled with a mass flow controller (Alicat Scientific). Titanium mesh coated with iridium was used as the counter electrode and Ag/AgCl as the reference electrode (saturated KCl, Gaossunion). The glassy carbon electrode loaded with the catalyst served as the working electrode. An electrochemical workstation (IVIUM CompactStat.e20250) was used to provide the external bias. The gas products were detected using an in-line multiple-gas analyser gas chromatography system (GC2060, Shanghai Ruimin Instrument Co., Ltd.). The liquid products were collected from the cathode and anode chambers after electrolysis and analyzed by headspace-gas chromatography (HS-GC). Applied cathode potentials after *iR*_cell_ compensation (*i* is the applied current and *R*_cell_ is the cell resistance) were converted to the RHE reference scale using *E*_RHE_ = *E*_Ag/AgCl_ + 0.197 V + 0.059 × pH; *R*_cell_ is determined by performing an electrochemical impedance spectroscopy measurement.

### OH adsorption

In order to clarify the change in surface structures, we have investigated the electrosorption of oxygenated species (e.g., O and/or OH) on the resulting OD-Cu particles surfaces to identify the exposed facets. The cyclic voltammograms were recorded using a customized H-type electrochemical cell and a potentiostat (Autolab PGSTAT 204, Metrohm). A Hg/HgO electrode (with saturated KCl solution as the filling solution, GaossUnion) and a graphite rod (GaossUnion) were used as the reference and counter electrode, respectively. A solution of 1 M KOH was used as the electrolyte. Ar was delivered to the cathode compartment at a constant rate of 20 sccm. The cathode and anode compartments were separated with an anion exchange membrane (Fumasep, FAA-3-PK-130). The low-index facets for *fcc* Cu crystals were labeled by comparing the CVs to those of single crystals shown in previous works^45,46^.

## Supplementary information

Supplementary Information

## Data Availability

The data supporting the findings of the study are available within the paper and its Supplementary Information.

## Code availability

The software code for LASP and the NN potentials used within the article are available from the website http://www.lasphub.com.
