# Supplementary material for: The nature of active sites for carbon dioxide electroreduction over oxide-derived copper catalysts
Source: Nat Commun. 2021 Jan 15;12:395. doi: 10.1038/s41467-020-20615-0 (PMC7810728; doi:10.1038/s41467-020-20615-0)
Supplement: Supplementary file 1 — Supplementary Information [file 41467_2020_20615_MOESM1_ESM.pdf]

Supplementary Materials for

The Nature of Active Sites for Carbon dioxide Electroreduction over Oxide-derived Copper Catalysts

Dongfang Cheng[#1,2], Zhi-Jian Zhao[#1,2], Gong Zhang[1,2], Piaoping Yang[1,2], Lulu Li[1,2], Hui Gao[1,2], Sihang Liu[1,2], Xin Chang[1,2], Sai Chen[1,2], Tuo Wang[1,2], Geoffrey A. Ozin[4], Zhipan Liu[5] and Jinlong Gong*[1,2,3]

[1]Key Laboratory for Green Chemical Technology of Ministry of Education, School of Chemical Engineering and Technology, Tianjin University, Tianjin 300072, China.

[2]Collaborative Innovation Center of Chemical Science and Engineering (Tianjin), Tianjin 300072, China.

[3]Joint School of National University of Singapore and Tianjin University, International Campus of Tianjin University, Binhai New City, Fuzhou 350207, China.

[4]Department of Chemistry, University of Toronto, Toronto, Canada.

[5]Collaborative Innovation Centre of Chemistry for Energy Material, Shanghai Key Laboratory of Molecular Catalysis and Innovative Materials, Key Laboratory of Computational Physical Science, Department of Chemistry, Fudan University, Shanghai, China.

* **Corresponding Author**

E-mail: jlgong@tju.edu.cn.

# These authors contributed equally to this work.

Supplementary Methods

LASP is a commercial software, which is developed by Prof. Zhipan Liu group at Fudan University. (http://www.lasphub.com/index.asp) Details are in WIREs Comput Mol Sci. 2019; e1415. General speaking, LASP code is developed for large-scale simulation of complex systems with neural network (NN) potential. By learning the first principles dataset of global potential energy surface (PES), which is obtained from stochastic surface walking (SSW) global optimization, global neural network potential is generated. PES exploration for complex materials can be achieved by combining the SSW method and NN potential. Apart from SSW-NN global optimization, the software implements standard interfaces to dock with other energy/force evaluation package and can perform tasks for PBS properties like molecular dynamics simulation.

The NN potential used in this calculation is provided by LASP software. The NN potential is generated by iterative self-learning of the plane-wave density functional theory (DFT) global PES data set generated from SSW exploration. There are two steps for generating NN potential. Firstly, based on DFT calculations using selected structures from the SSW simulation, global data-set is generated. Next, NN potential is fitted based on high dimensional neural network scheme introduced by Behler and Parinello. These steps are iteratively performed until the NN potential is transferable and robust enough to describe the global PES. The procedure is briefly summarized below.

At first, the global data set was built iteratively during the self-learning of the NN potential. The initial data of the global data set came from the DFT-based SSW simulation and all the other data were taken from the NN-based SSW PES exploration. To cover all the likely compositions of CuO and CuCHO, SSW simulations were carried out for different structures (which included bulk, layer and cluster), compositions and atom number per unit cell. Overall, these SSW simulations generated more than 10^7 structures on the PES of CuO and CuCHO. The final global data set computed from the high-accuracy DFT calculation contained 17103 structures

and 68440 for CuO and CuCHO system, as detailed in Supplementary Tables 1 and 2.

Then, the NN potential was generated using the method as introduced by Behler and Parinello. To pursue a high accuracy for PES, a large set of power-type structure descriptors was adopted. For Cu-O NN potential, we used 147 descriptors for every element, including 84 two-body, 57 three-body and 6 four-body descriptors, and compatibly; the network utilized was also large as it involved three-hidden layers (147-50-50-1 net). For Cu-C-H-O NN potential, we used 274 descriptors for every element, including 100 two-body, 164 three-body and 10 four-body descriptors, and compatibly; the network utilized was also large as it involved three-hidden layers (274-50-50-1 net). Min-max scaling was utilized to normalization the training data sets. Hyperbolic tangent activation functions were used for the hidden layers, whereas a linear transformation was applied to the output layer of all the networks. The limited memory Broyden–Fletcher–Goldfarb–Shanno method was used to minimize the loss function to match the DFT energy, force and stress. The final energy and force criteria of the r.m.s. errors were around 4.495 meV atom^{-1} and 0.094 eV Å^{-1} respectively for Cu-O system and 4.604 meV atom^{-1} and 0.106 eV Å^{-1} respectively for Cu-C-H-O system.

Supplementary Table 1 and 2 are the structure information in the first principles global dataset of Cu-O and Cu-C-H-O systems.

Supplementary Table 1 | Structure information in the first principles global dataset of Cu-O system. Listed data are the number of the structures in the global dataset, as distinguished by the chemical formula, the number of atoms per cell (N_{atom}), the type of structures (cluster, bulk and layer).

Species	N_{atom}	cluster	layer	bulk	total
Cu14	14	0	3	33	36
Cu15	15	87	6	1000	1093
Cu16	16	1155	71	5931	7157

Cu17	17	0	0	26	26
Cu28	28	0	0	35	35
Cu29	29	0	25	0	25
Cu30	30	0	55	32	87
Cu31	31	0	0	79	79
Cu32	32	0	2	95	97
O1-Cu16	17	0	6	34	40
O2-Cu16	18	0	6	53	59
O3-Cu16	19	0	3	39	42
O4	4	0	94	0	94
O4-Cu16	20	0	6	54	60
O6-Cu4	10	0	0	2955	2955
O6-Cu16	22	0	1	137	138
O7-Cu8	15	0	0	1335	1335
O8-Cu8	16	0	0	2566	2566
O8-Cu16	24	0	18	67	85
O10-Cu16	26	0	1	43	44
O11	11	0	478	146	624
O11-Cu16	27	0	0	13	13
O12-Cu16	28	0	3	50	53
O14-Cu16	30	0	1	44	45
O15-Cu16	31	0	1	46	47
O16-Cu16	32	0	19	26	45
O22-Cu16	38	0	0	205	205
O24-Cu16	40	0	0	18	18
total	--	1242	799	15062	17103

Supplementary Table 2 | Structure information in the first principles global dataset of Cu-C-H-O system. Listed data are the number of the structures in the global dataset, as distinguished by the chemical formula, the number of atoms per cell (Natom), the type of structures (cluster, bulk and layer).

Species	N_{atom}	cluster	layer	bulk	total
H2-O1-Cu27	30	0	450	0	450
H2-C1-O2-Cu11	16	0	4	23	27
H2-C1-O2-Cu12	17	0	15453	785	16238
H2-C1-O2-Cu16	21	0	635	0	635
H2-C1-O2-Cu27	32	0	651	0	651
H2-C2-O3-Cu27	34	0	1474	0	1474
H2-C2-O3-Cu36	43	0	1947	0	1947
H2-C3-O4-Cu33	42	0	713	0	713
H2-C3-O4-Cu35	44	0	743	0	743
H2-C3-O4-Cu36	45	0	1508	0	1508
H3-C1-O2-Cu18	24	0	478	0	478
H3-C2-O2-Cu31	38	0	6	0	6
H3-C2-O2-Cu32	39	0	6	0	6
H3-C2-O2-Cu36	43	0	805	0	805
H3-C2-O3-Cu23	31	0	55	0	55
H3-C2-O3-Cu24	32	0	217	5	222
H3-C2-O3-Cu27	35	0	74	0	74
H4-C1-O1-Cu12	18	0	967	3	970
H4-C1-O3-Cu27	35	0	5936	0	5936
H4-C2-O2-Cu27	35	0	71	0	71
H4-C2-O2-Cu33	41	0	577	0	577
H4-C2-O2-Cu36	44	0	582	0	582
H4-C2-O3-Cu36	45	0	239	0	239

H4-C2-O4-Cu11	21	0	15	22	37
H4-C2-O4-Cu12	22	0	227	6105	6332
H4-C2-O4-Cu24	34	0	274	32	306
H4-C2-O4-Cu26	36	0	45	0	45
H4-C2-O4-Cu27	37	0	2566	1	2567
H4-C2-O4-Cu32	42	0	109	0	109
H4-C2-O4-Cu36	46	0	1753	0	1753
H4-C3-O5-Cu36	48	0	44	0	44
H5-C1-O2-Cu23	31	0	10	0	10
H6-C1-O1-Cu27	35	0	570	0	570
H6-C1-O2-Cu27	36	0	3245	0	3245
H6-C1-O3-Cu12	22	0	108	2528	2636
H6-C2-O3-Cu36	47	0	2547	0	2547
H8-C1-O2-Cu27	38	0	20	0	20
H8-C2-O2-Cu23	35	0	34	8	42
H8-C2-O2-Cu24	36	0	71	27	98
H12-C2-O4-Cu12	30	0	93	7024	7117
H14-O7	21	0	1	808	809
H16-O8	24	0	14	3955	3969
H30-O15	45	124	4	94	222
H48-O24-Cu24	96	0	47	0	47
H54-C2-O26-Cu22	104	0	0	77	77
H55-C1-O26-Cu22	104	0	0	72	72
H56-C1-O25-Cu22	104	0	0	74	74
H56-C1-O26-Cu22	105	0	0	74	74
H57-C1-O26-Cu22	106	0	0	74	74
H94-O42-Cu64	200	0	58	0	58
H95-C1-O44-Cu63	203	0	93	2	95
H95-C1-O44-Cu64	204	0	112	1	113

H95-C1-O45-Cu62	203	0	114	2	116
H95-C1-O45-Cu63	204	0	125	1	126
H95-C1-O45-Cu64	205	0	102	0	102
H95-C2-O46-Cu62	205	0	81	0	81
H96-O43-Cu64	203	0	88	0	88
H96-C1-O45-Cu60	202	0	77	2	79
H96-C1-O45-Cu62	204	0	91	0	91
H96-C2-O45-Cu62	205	0	90	0	90
H96-C2-O46-Cu61	205	0	95	3	98
total	--	124	46514	21802	68440

Furthermore, to validate the accuracy of the NN potential predicted oxygen vacancy formation energy (Eov), *CO(E(*CO)) and *COCO(E(*COCO)) intermediates adsorption energies, we conducted high-cost DFT single-point calculations on our large OD-Cu models to compare with the NN results. The RMS error for Eov, E(*CO) and E(*COCO) are 0.12eV, 0.08eV, 0.13eV, repectively. Details are given in Supplementary Tables 3-5.

Supplementary Table 3 | The comparison of Cu-O NN potential predicted oxygen vacancy formation energy and that from DFT calculation.

	$E_{NN\text{-}potential}$/eV	E_{DFT}/eV	ΔE/eV
1	7.26	7.40	-0.14
2	6.64	6.78	-0.14
3	6.95	6.86	0.09
4	7.21	7.37	-0.16
5	7.80	7.76	0.04
6	7.38	7.65	-0.27
7	7.17	7.10	0.07

8	7.61	7.64	-0.04
9	7.13	7.31	-0.18
10	7.30	7.35	-0.05
11	7.62	7.58	0.04
12	6.60	6.66	-0.06
13	7.87	7.82	0.04
14	7.87	8.00	-0.13
15	7.92	8.01	-0.09
RMSE			0.12

Supplementary Table 4 | The comparison of Cu-C-H-O NN potential predicted CO* adsorption energy and that from DFT calculation.

	$E_{NN\text{-}potential}$/eV	E_{DFT}/eV	ΔE/eV
1	-0.71	-0.77	0.06
2	-0.77	-0.74	-0.03
3	-0.84	-0.90	0.06
4	-0.68	-0.67	-0.01
5	-0.81	-0.76	-0.05
6	-0.62	-0.74	0.12
7	-0.52	-0.67	0.15
8	-0.90	-0.76	-0.14
9	-0.64	-0.68	0.03
10	-0.82	-0.84	0.02
11	-0.86	-0.79	-0.07
12	-0.65	-0.57	-0.08
13	-0.53	-0.52	-0.01
14	-0.48	-0.47	-0.01
15	-0.46	-0.34	-0.11

16	-0.65	-0.57	-0.08
17	-0.63	-0.52	-0.11
18	-0.60	-0.65	0.05
19	-0.98	-0.94	-0.05
20	-0.59	-0.67	0.08
21	-0.95	-0.99	0.04
22	-0.95	-0.88	-0.07
23	-0.96	-0.78	-0.19
24	-0.60	-0.67	0.07
25	-0.98	-0.90	-0.08
26	-0.81	-0.75	-0.07
27	-0.41	-0.34	-0.06
28	-0.91	-0.71	-0.20
29	-0.68	-0.73	0.05
30	-0.91	-0.79	-0.12
31	-0.93	-0.89	-0.04
32	-0.89	-0.75	-0.14
33	-0.65	-0.67	0.02
34	-0.86	-0.73	-0.13
35	-0.84	-0.72	-0.12
36	-0.86	-0.91	0.06
37	-0.85	-0.80	-0.05
38	-0.85	-0.72	-0.13
39	-0.81	-0.82	0.01
40	-0.70	-0.66	-0.04
41	-0.85	-0.84	-0.01
42	-0.88	-0.85	-0.03
43	-0.88	-0.75	-0.13
44	-0.82	-0.80	-0.01

45	-0.82	-0.80	-0.02
46	-0.79	-0.80	0.01
47	-0.76	-0.65	-0.11
48	-0.65	-0.57	-0.08
49	-0.70	-0.75	0.05
50	-0.67	-0.62	-0.04
RMSE			0.08

Supplementary Table 5 | The comparison of Cu-C-H-O NN potential predicted *COCO adsorption energy and that from DFT calculation.

	$E_{NN\text{-}potential}$/eV	E_{DFT}/eV	ΔE/eV
1	-0.15	0.00	-0.15
2	-0.23	-0.23	0.00
3	0.19	0.14	0.06
4	-0.37	-0.35	-0.02
5	-0.01	0.10	-0.12
6	0.15	0.30	-0.15
7	-0.30	-0.02	-0.27
8	-0.12	-0.04	-0.07
9	-0.33	-0.31	-0.03
10	-0.18	-0.27	0.09
11	-0.09	-0.01	-0.08
12	-0.15	-0.28	0.13
13	0.18	-0.05	0.23
14	0.13	-0.05	0.17
15	-0.02	-0.09	0.07
RMSE			0.13

OD-Cu-1

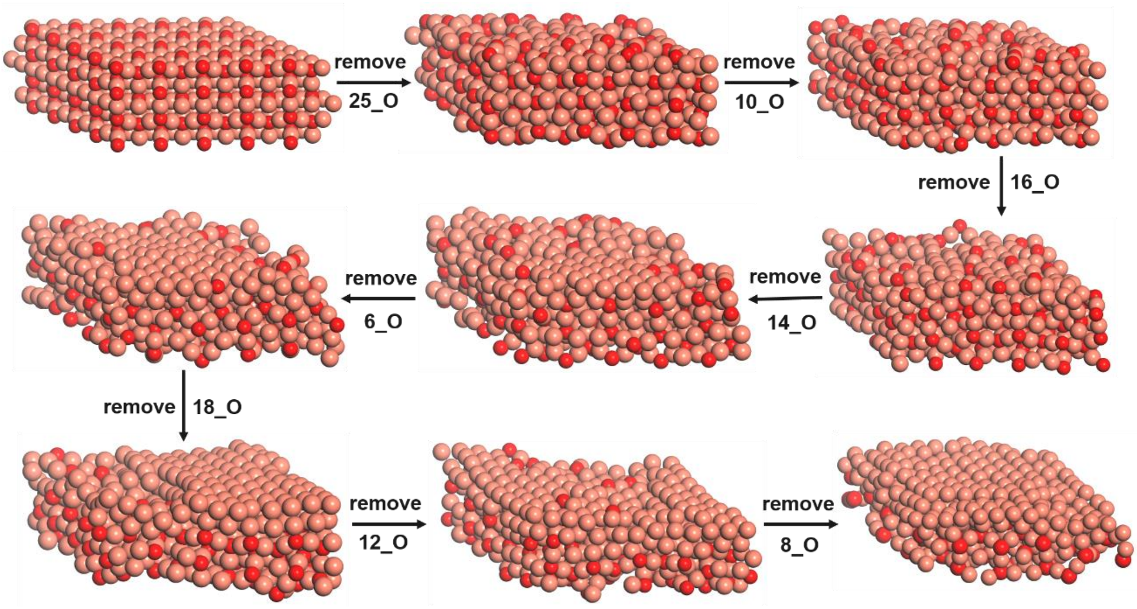

Supplementary Fig. 1 | Schematic diagram of reduction process. Structures of every stage in the reduction process of Cu_2O to OD-Cu-1.

OD-Cu-1

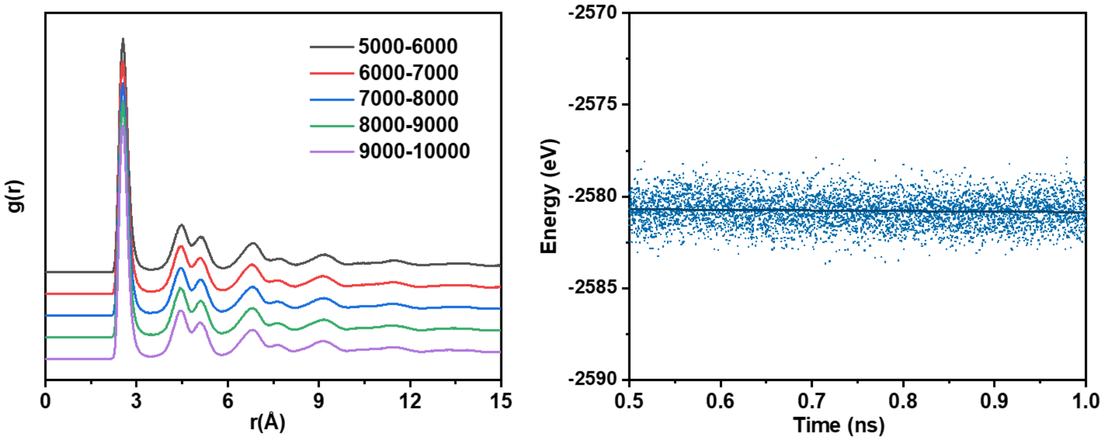

Supplementary Fig. 2 | Radial distribution function and energy profiles in the final 0.5ns MD simulation. The surface structure tends to balance after 0.5ns simulation according to the radial distribution function of surface Cu atoms and energy profile of NN-MD simulation for the whole system. Meanwhile, in the final 0.5ns, nearly all the atoms oscillated near their equilibrium position without obvious diffusion.

OD-Cu-2

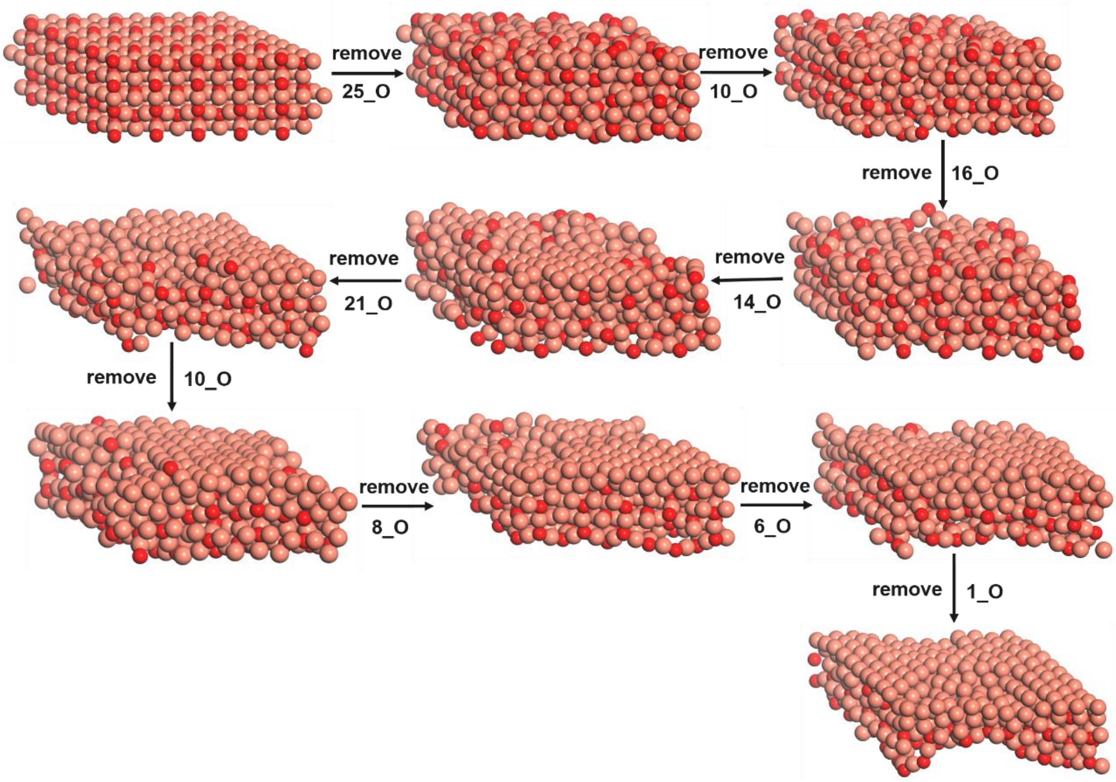

Supplementary Fig. 3 | Schematic diagram of reduction process. Structures of every stage in the reduction process of Cu_2O to OD-Cu-2.

OD-Cu-2

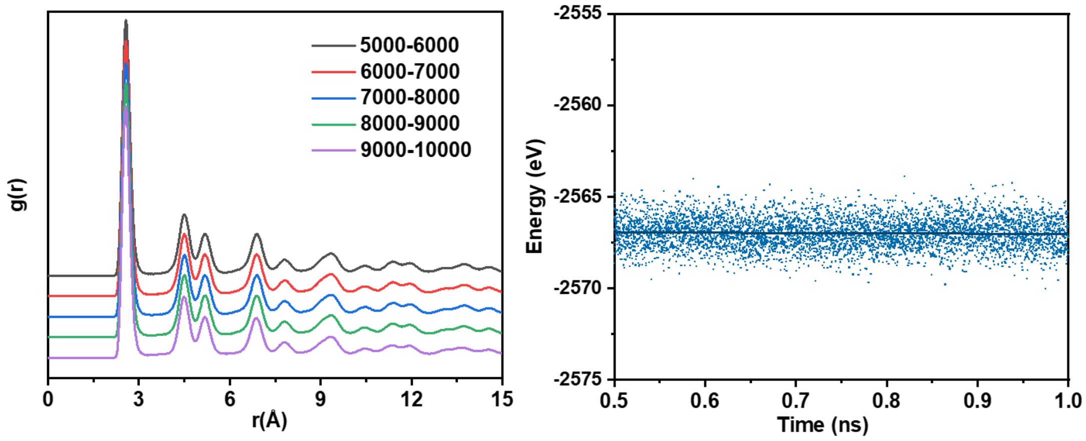

Supplementary Fig. 4 | Radial distribution function and energy profiles in the final 0.5ns MD simulation. The surface structure tends to balance after 0.5ns simulation according to the radial distribution function of surface Cu atoms and energy profile of NN-MD simulation for the whole system. Meanwhile, in the final 0.5ns, nearly all the atoms oscillated near their equilibrium position without obvious diffusion.

OD-Cu-3

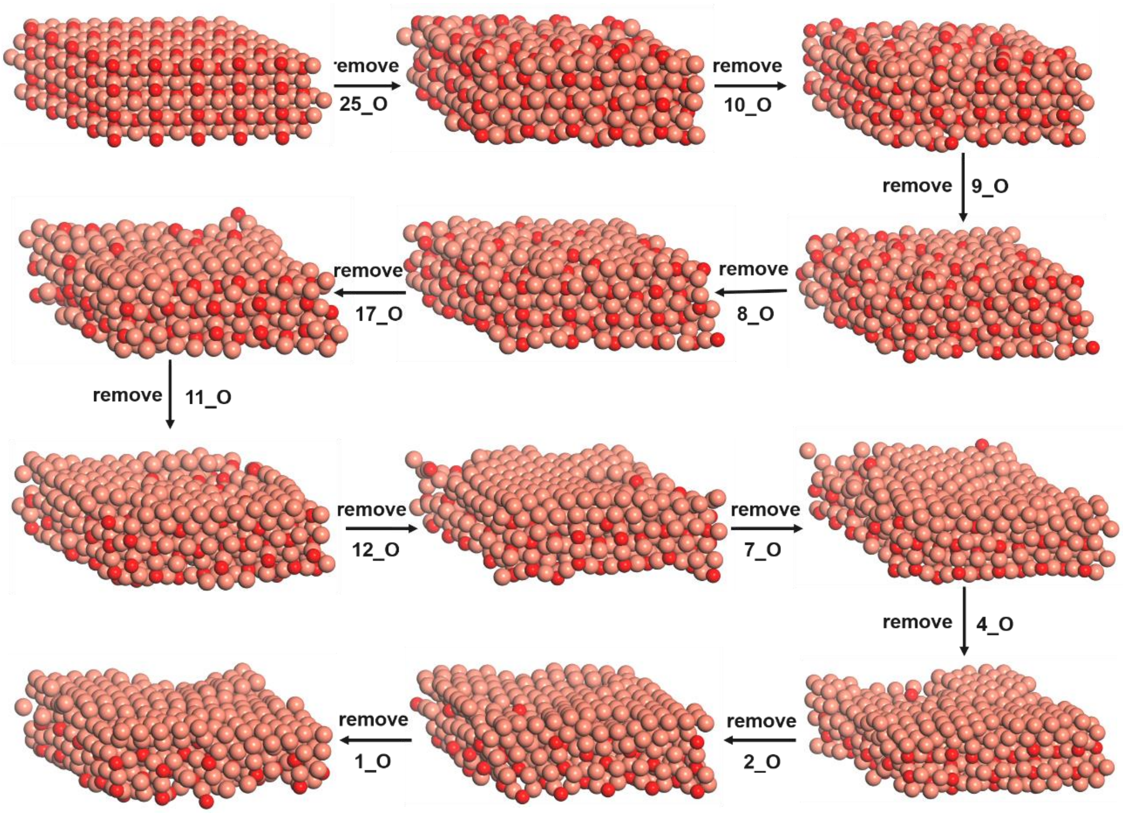

Supplementary Fig. 5 | Schematic diagram of reduction process. Structures of every stage in the reduction process of Cu_2O to OD-Cu-3.

OD-Cu-3

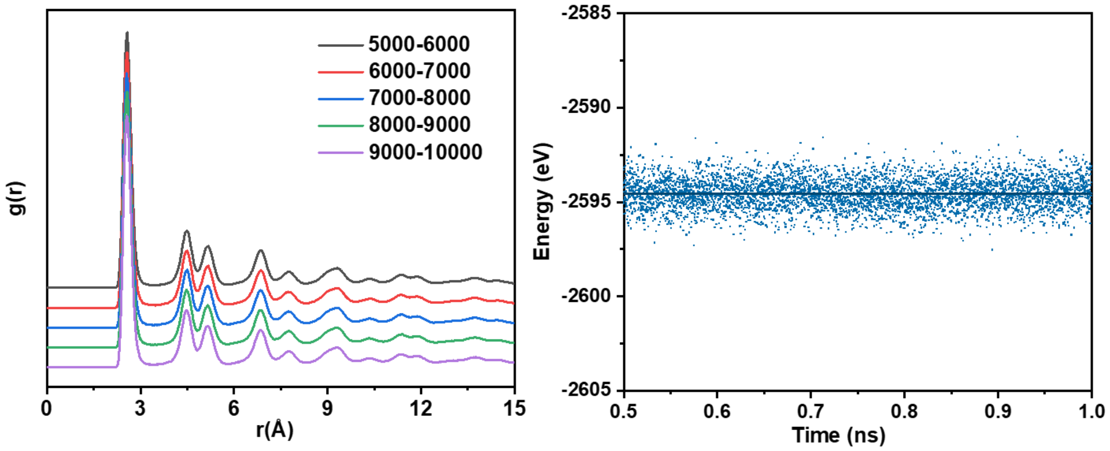

Supplementary Fig. 6 | Radial distribution function and energy profiles in the final 0.5ns MD simulation. The surface structure tends to balance after 0.5ns simulation according to the radial distribution function of surface Cu atoms and energy profile of NN-MD simulation for the whole system. Meanwhile, in the final 0.5ns, nearly all the atoms oscillated near their equilibrium position without obvious diffusion.

OD-Cu-4

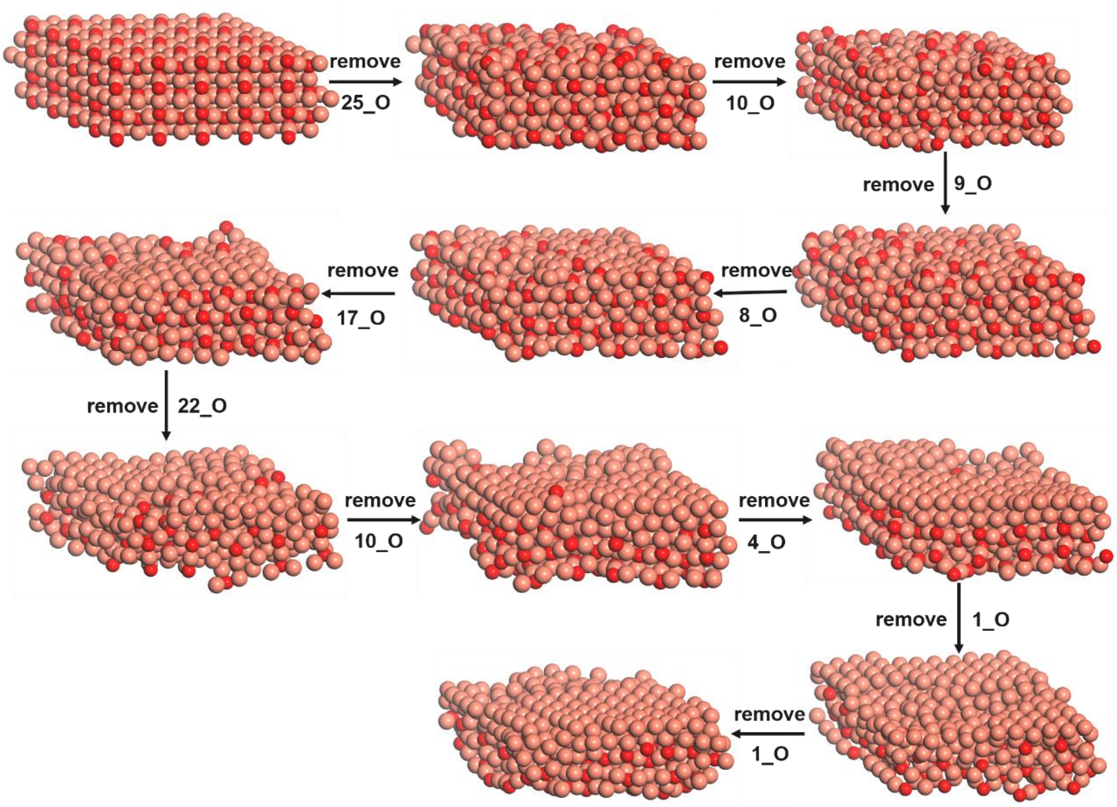

Supplementary Fig. 7 | Schematic diagram of reduction process. Structures of every stage in the reduction process of Cu_2O to OD-Cu-4.

OD-Cu-4

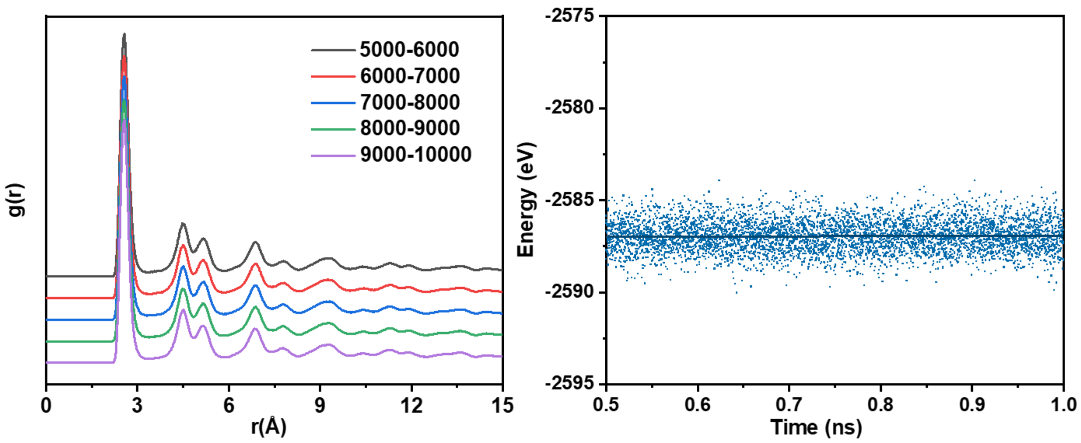

Supplementary Fig. 8 | Radial distribution function and energy profiles in the final 0.5ns MD simulation. The surface structure tends to balance after 0.5ns simulation according to the radial distribution function of surface Cu atoms and energy profile of NN-MD simulation for the whole system. Meanwhile, in the final 0.5ns, nearly all the atoms oscillated near their equilibrium position without obvious diffusion.

OD-Cu-5

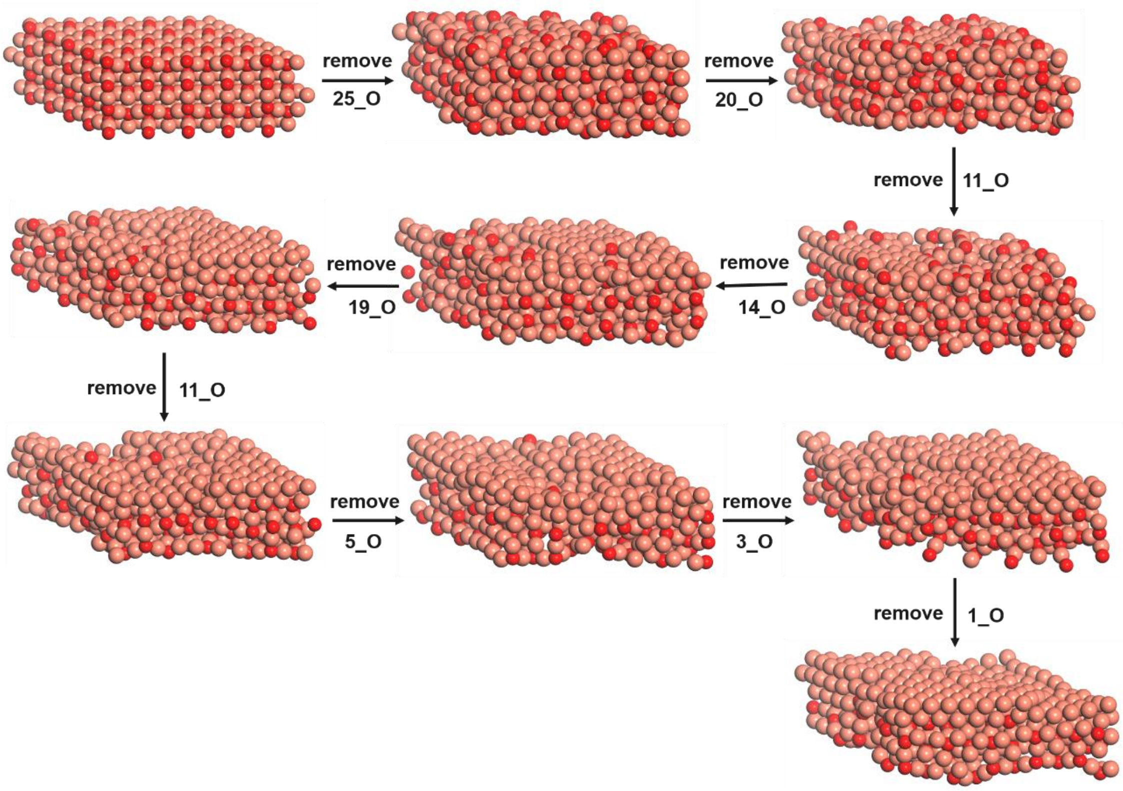

Supplementary Fig. 9 | Schematic diagram of reduction process. Structures of every stage in the reduction process of Cu_2O to OD-Cu-5.

OD-Cu-5

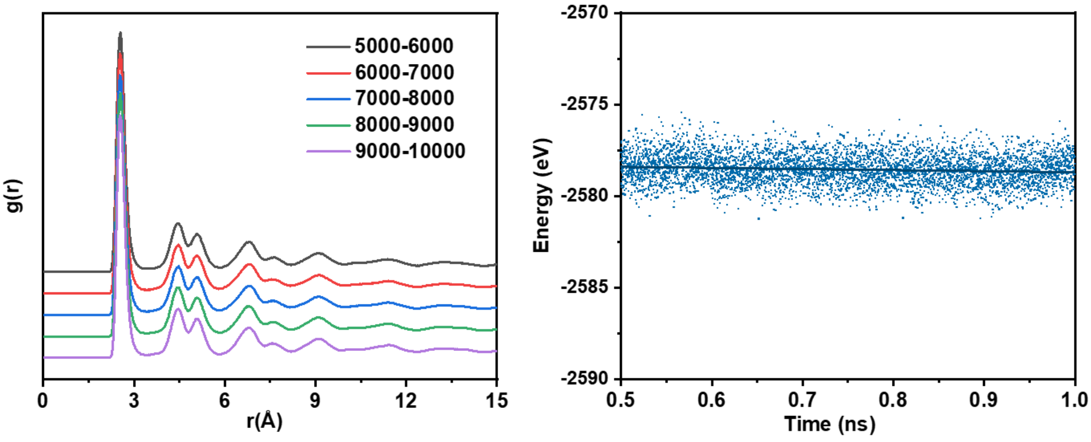

Supplementary Fig. 10 | Radial distribution function and energy profiles in the final 0.5ns MD simulation. The surface structure tends to balance after 0.5ns simulation according to the radial distribution function of surface Cu atoms and energy profile of NN-MD simulation for the whole system. Meanwhile, in the final 0.5ns, nearly all the atoms oscillated near their equilibrium position without obvious diffusion.

OD-Cu-6

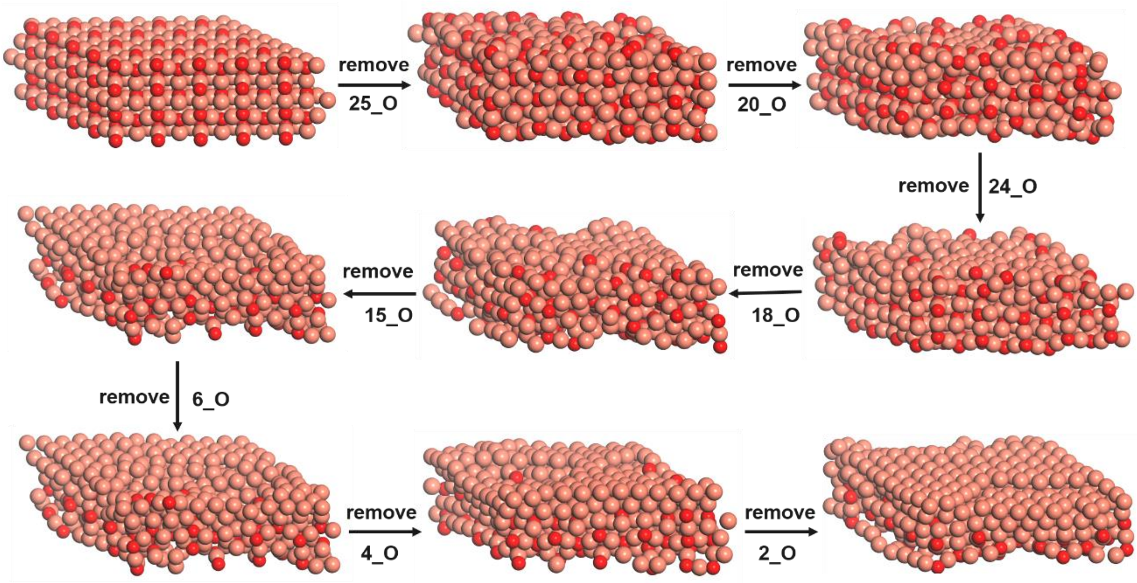

Supplementary Fig. 11 | Schematic diagram of reduction process. Structures of every stage in the reduction process of Cu_2O to OD-Cu-6.

OD-Cu-6

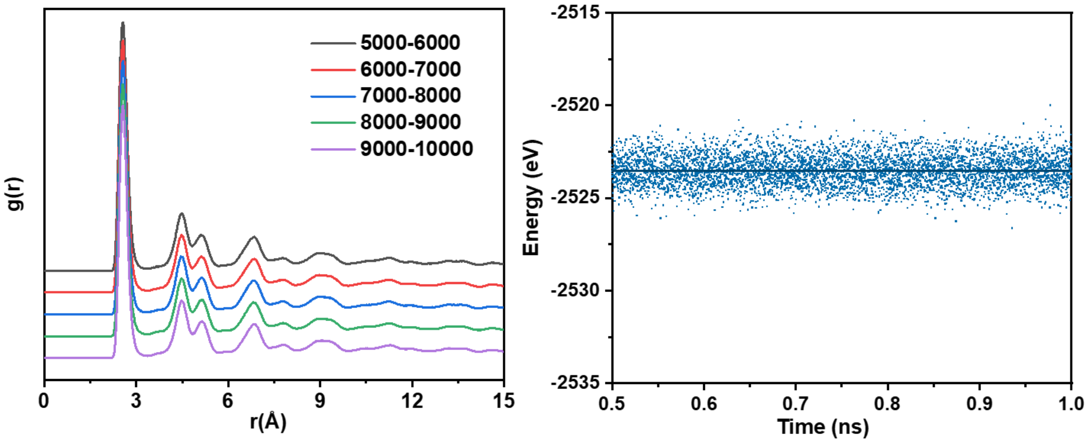

Supplementary Fig. 12 | Radial distribution function and energy profiles in the final 0.5ns MD simulation. The surface structure tends to balance after 0.5ns simulation according to the radial distribution function of surface Cu atoms and energy profile of NN-MD simulation for the whole system. Meanwhile, in the final 0.5ns, nearly all the atoms oscillated near their equilibrium position without obvious diffusion.

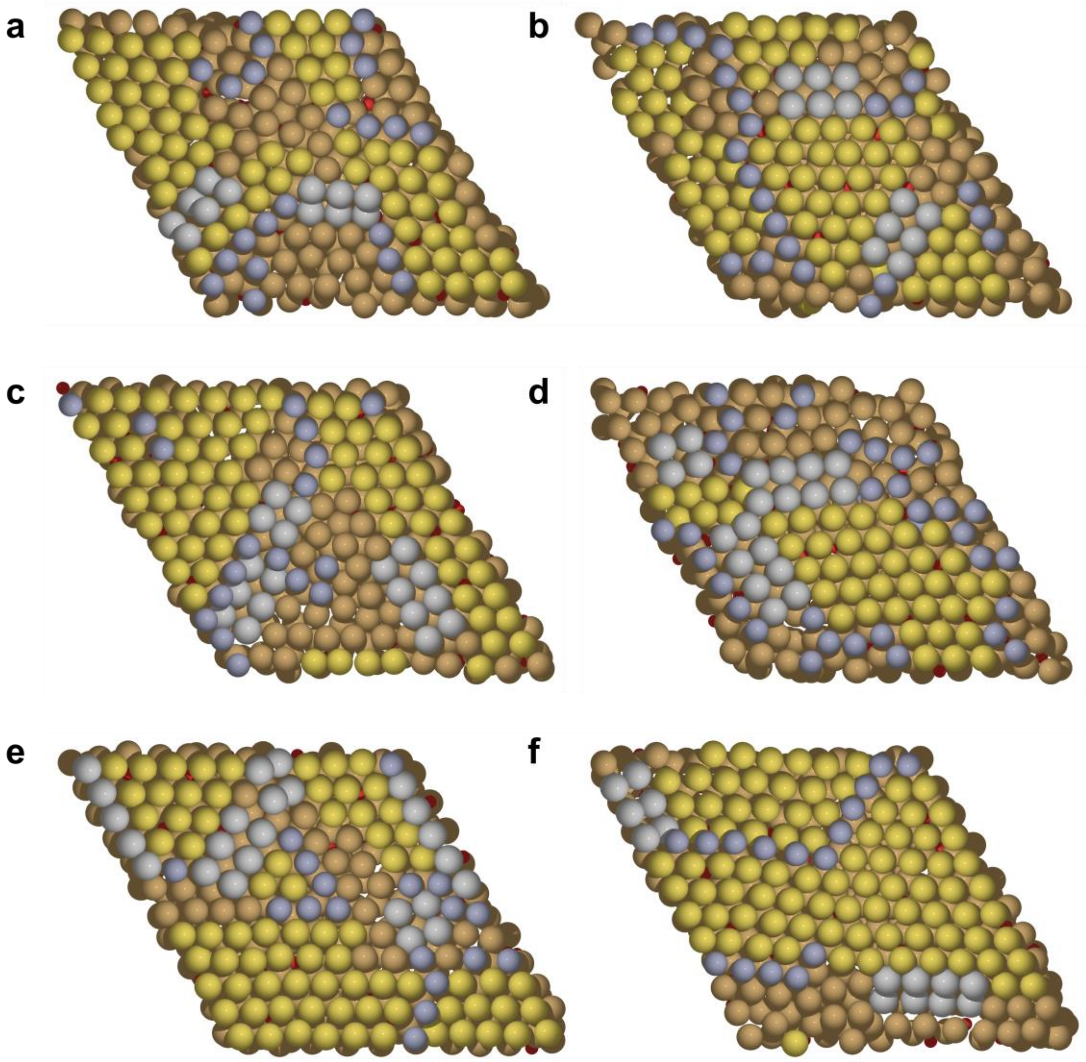

Supplementary Fig. 13 | Surface structure of OD-Cu. a, OD-Cu-1 **b,** OD-Cu-2 **c,** OD-Cu-3 **d,** OD-Cu-4 **e,** OD-Cu-5 **f,** OD-Cu-6. Grey, blue and yellow balls stand for square-like, step sites and 111 respectively.

The local optimized final structures of the 1ns NN-MD simulation are proposed to be OD-Cu surface structures. The upper two layers have become fully metallic copper, while O atoms are all trapped in the third layer and below. In our simulated OD-Cu surface structures, (111) facet dominates. Besides, there are some obvious step sites and a few defects like grain boundaries and vacancies.

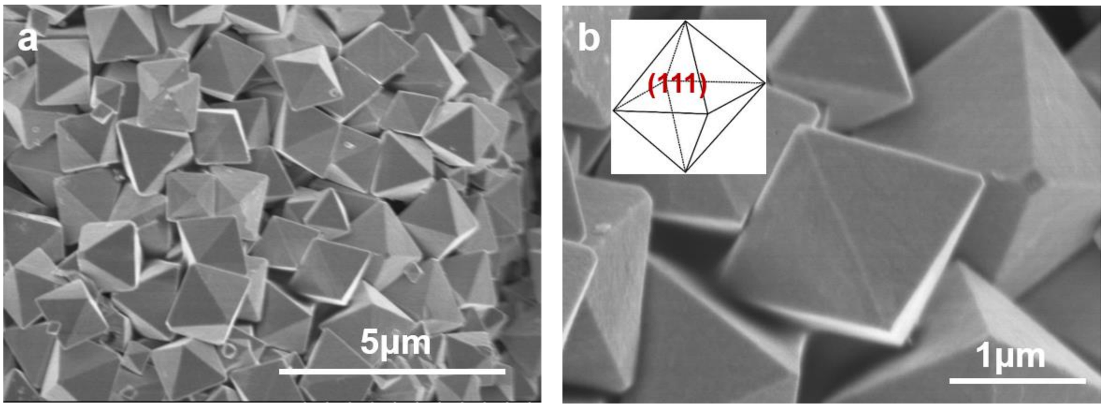

Supplementary Fig. 14 | Physical characterizations of Cu_2O. (a-b) SEM images of Cu_2O octahedron at different magnifications.

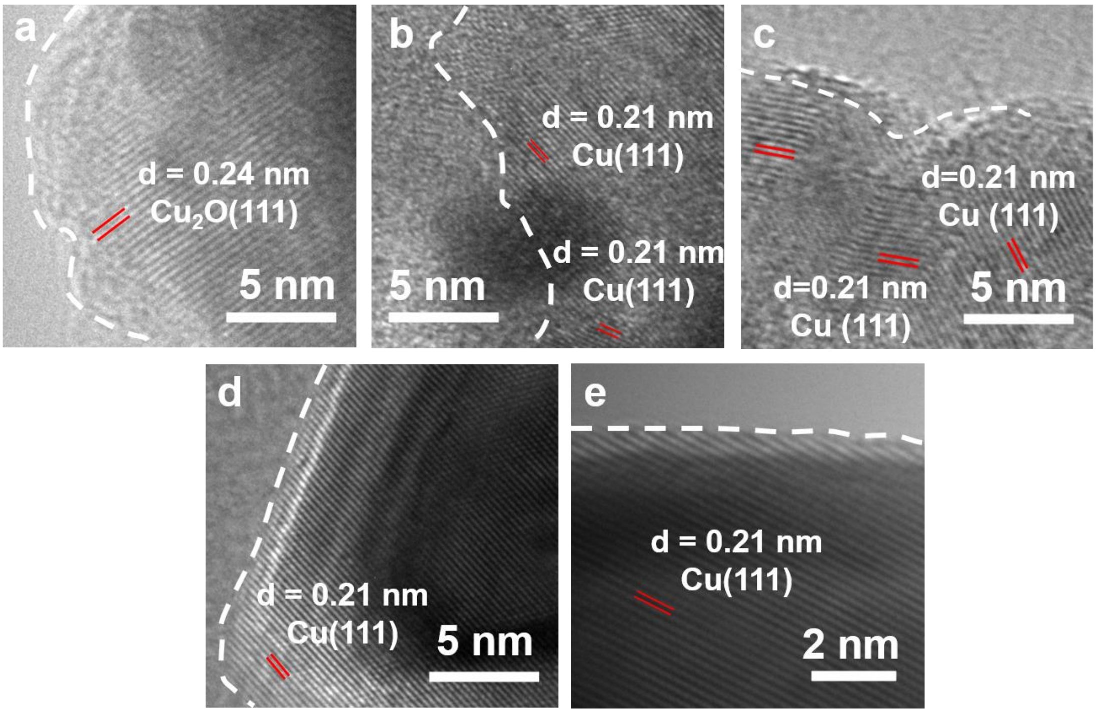

Supplementary Fig. 15 | TEM images a, Cu_2O **b,** OD-Cu **c,** after annealing at 450K **d,** after annealing at 500K **e,** after annealing at 650K

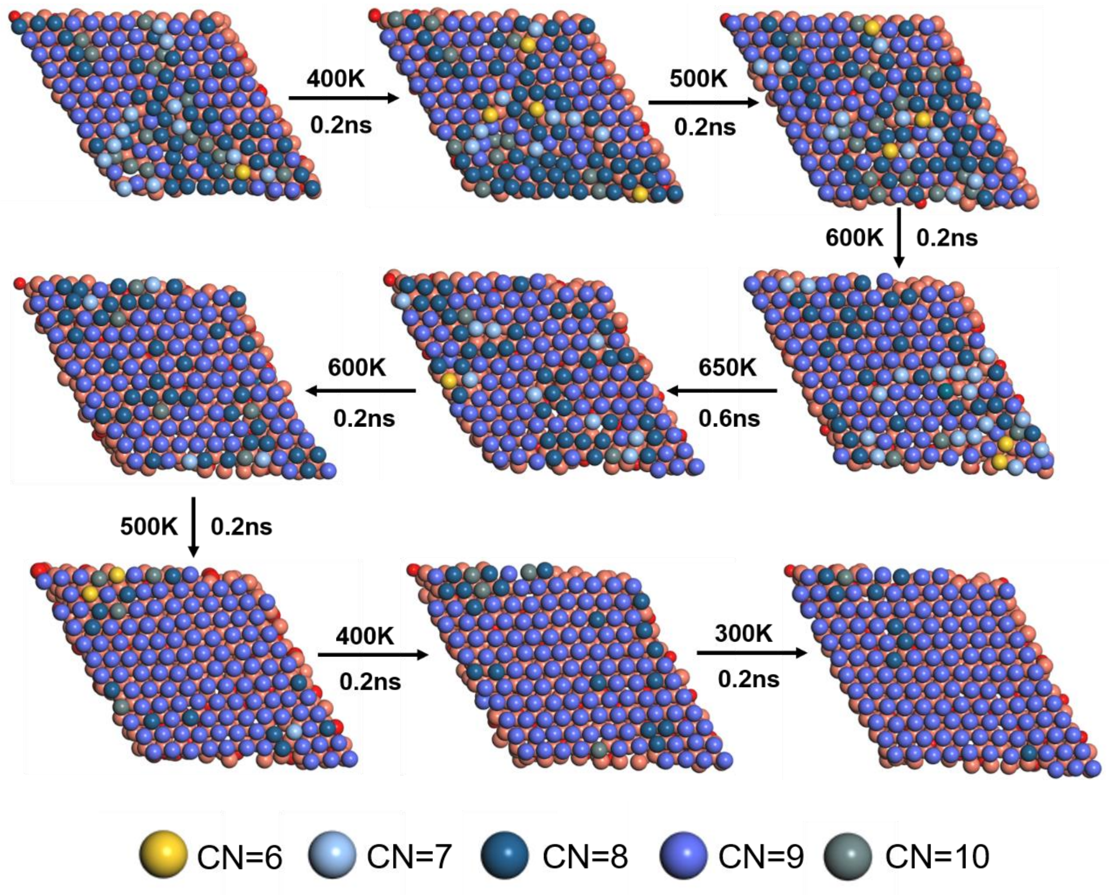

Supplementary Fig. 16 | Annealing process of OD-Cu-2 at 650K. Surface structures of every stage.

After annealing, the surface has been smoother. Step sites and some defects have been erased in this process.

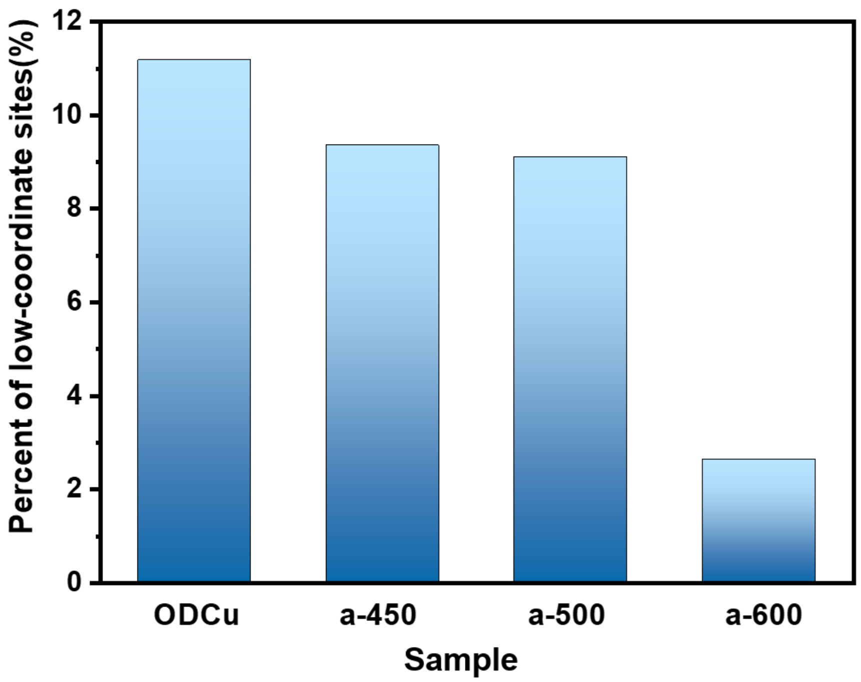

Supplementary Fig. 17 | Surface structures of OD-Cu. The percentage of low-coordinate sites on different samples.

The increase in annealing temperature results in the decreased proportion of low coordination sites on the surface

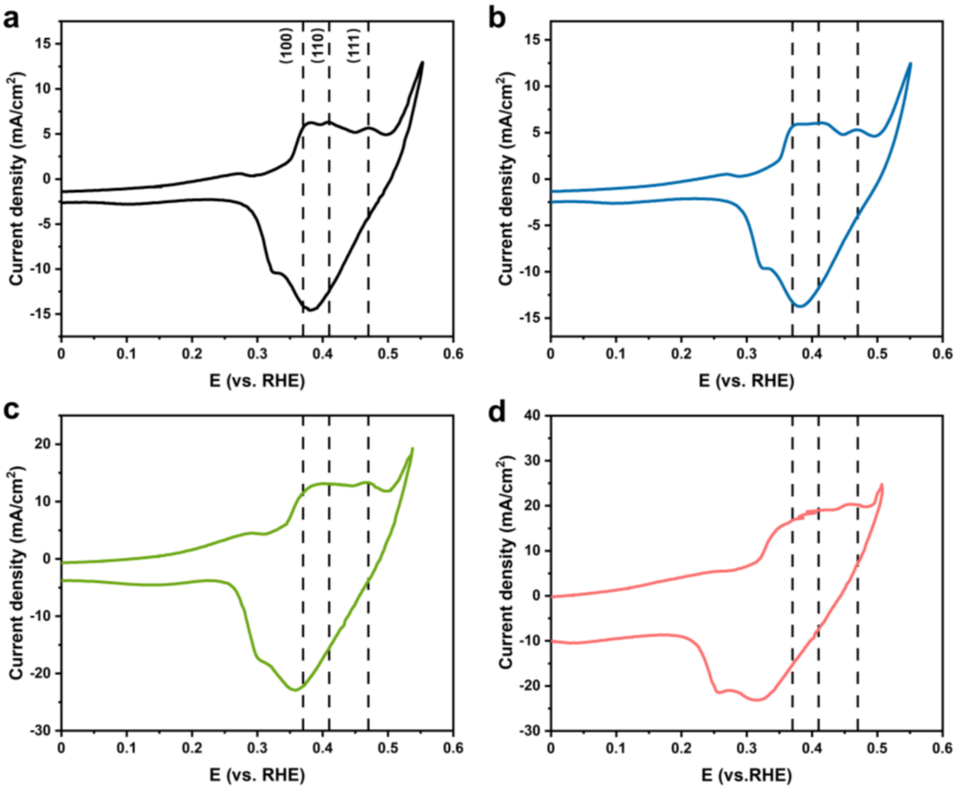

Supplementary Fig. 18 | OH-adsorption. CV curves collected in Ar-saturated 1M KOH for **a,** OD-Cu b, after annealing at 450K **c,** after annealing at 500K **d,** after annealing at 650K

As the annealing temperature increases, (111) becomes the dominent facet of the surface, while the proportion of the other facets becomes smaller, which is consistent with our NN-MD simulation results (Supplementary Fig. 16,17).

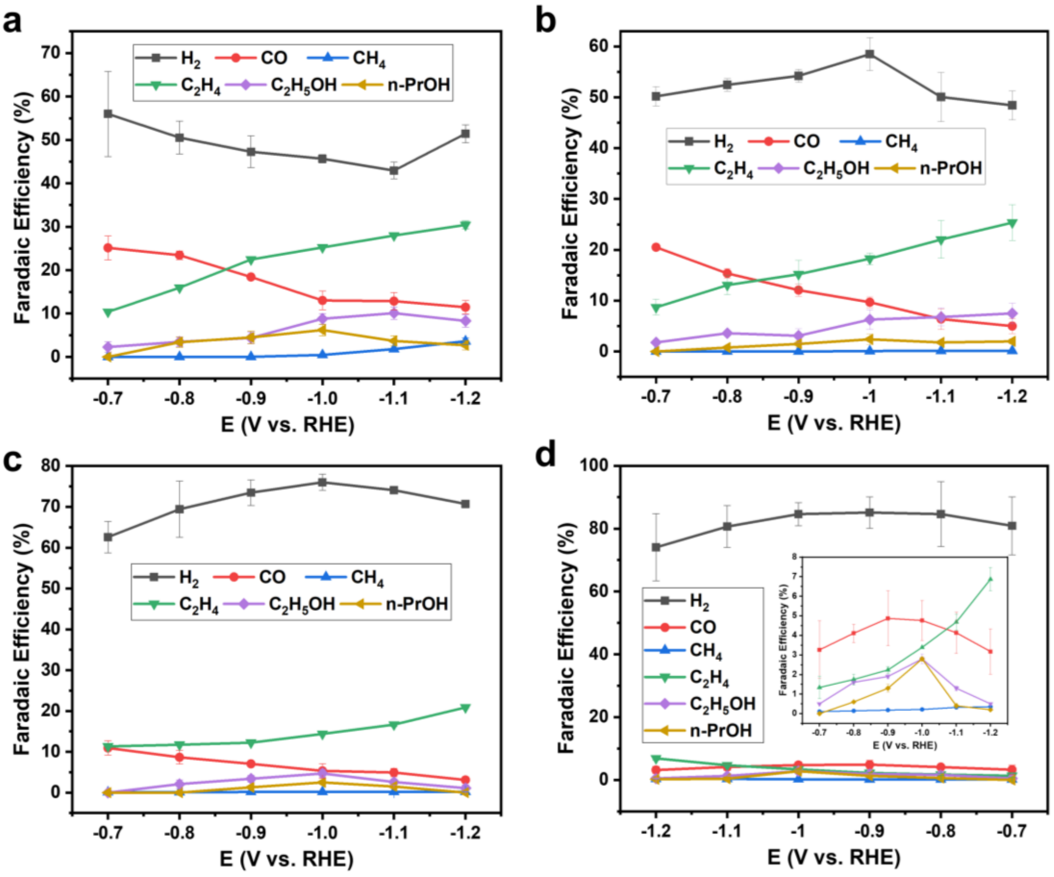

Supplementary Fig. 19 | Faradaic efficiencies (FE) for different products produced under a range of potentials a, OD-Cu **b,** a-450 **c,** a-500 **d,** a-650 The error bars represent the standard deviation from three independent measurements.

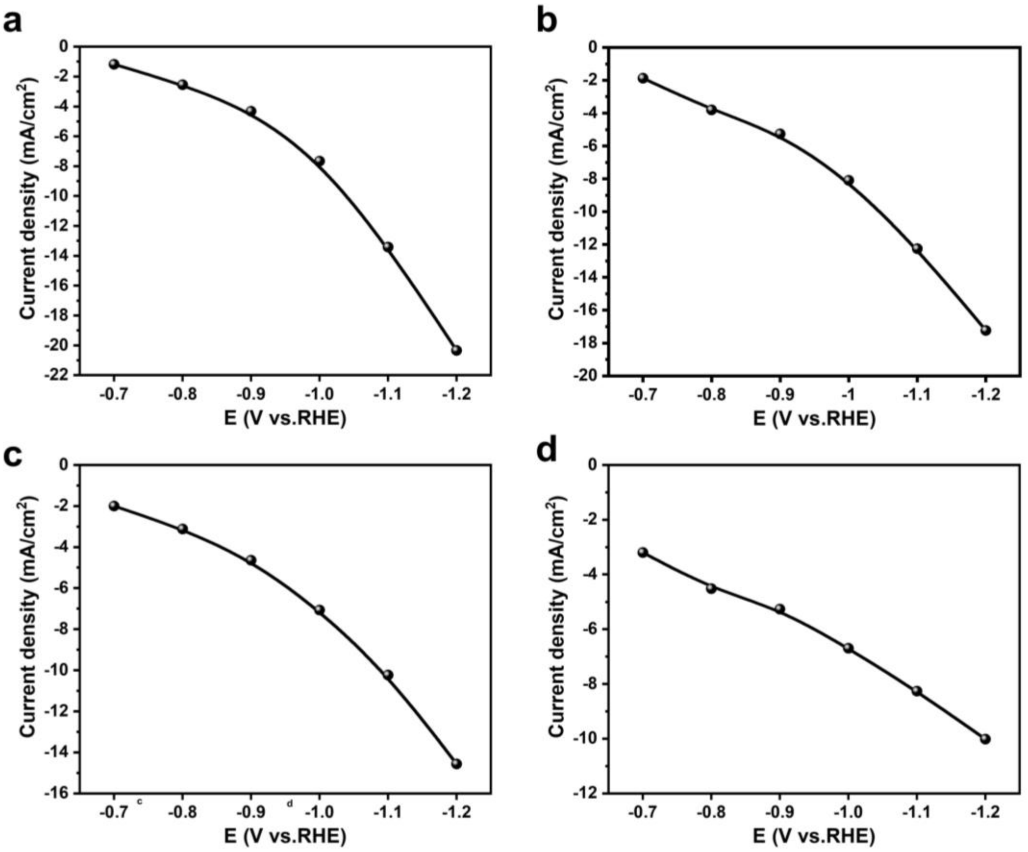

Supplementary Fig. 20 | Current Densities under a range of potentials a, OD-Cu **b,** a-450 **c,** a-500 **d,** a-650

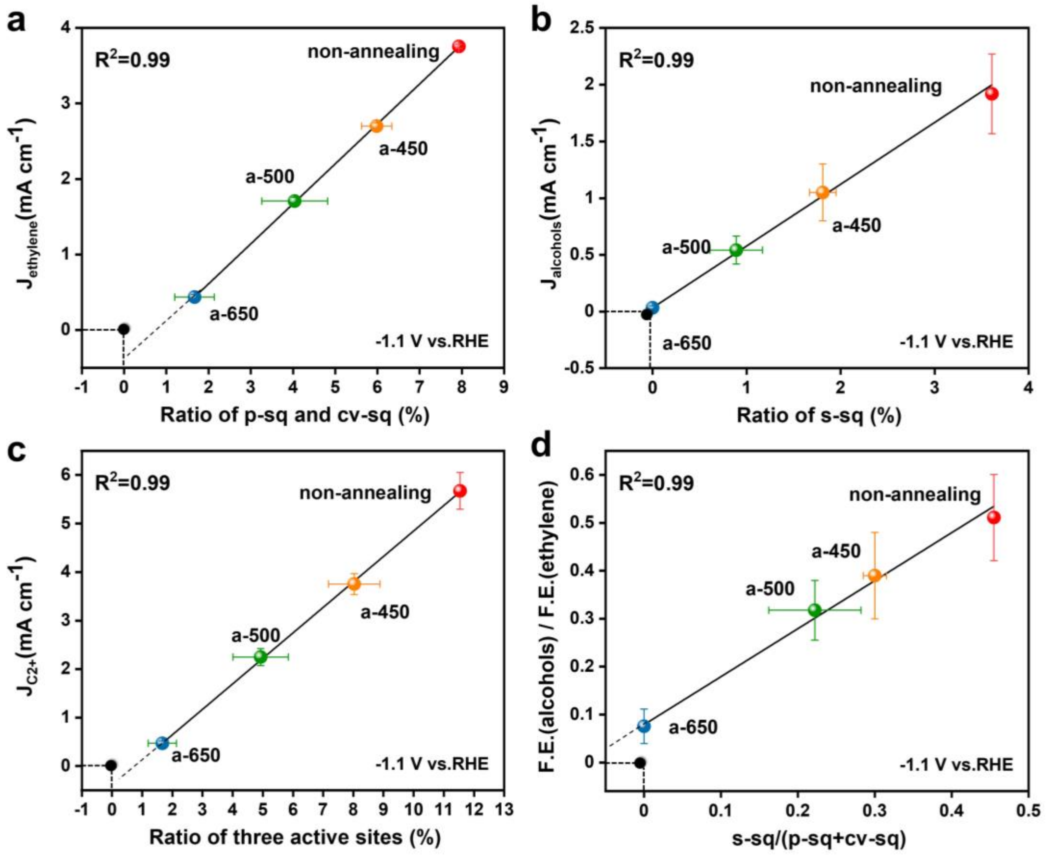

Supplementary Fig. 21 | Verification of active sites via thermal annealing process. The correlation between the current density of **a,** ethylene and the ratios of planar-square and convex-square sites **b,** alcohols and the ratios of step-square sites **c,** C_{2+} products and the ratios of all three active sites. **d,** Alcohols/ethylene ratios as the function of the ratios of step-square sites to the sum of planar-square and convex-square sites at -1.1V vs. RHE. The error bars represent the standard deviation from three independent measurements.

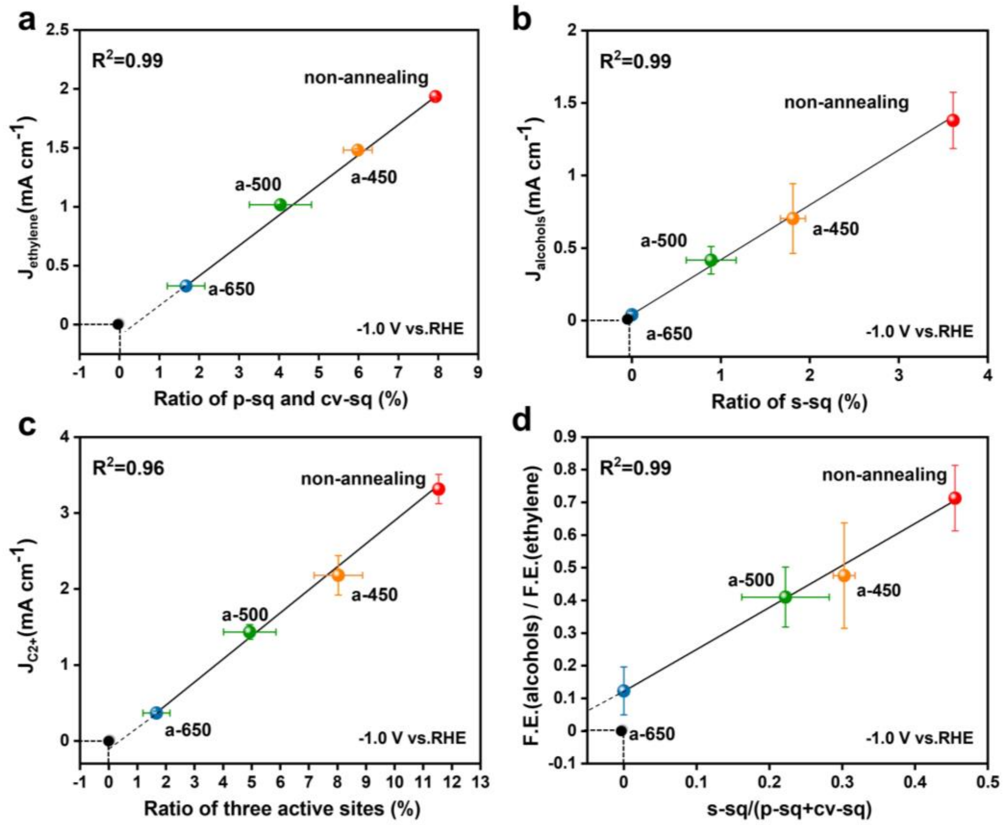

Supplementary Fig. 22 | Verification of active sites via thermal annealing process. The correlation between the current density of **a,** ethylene and the ratios of planar-square and convex-square sites **b,** alcohols and the ratios of step-square sites **c,** C_{2+} products and the ratios of all three active sites. **d,** Alcohols/ethylene ratios as the function of the ratios of step-square sites to the sum of planar-square and convex-square sites at -1.0V vs. RHE. The error bars represent the standard deviation from three independent measurements.

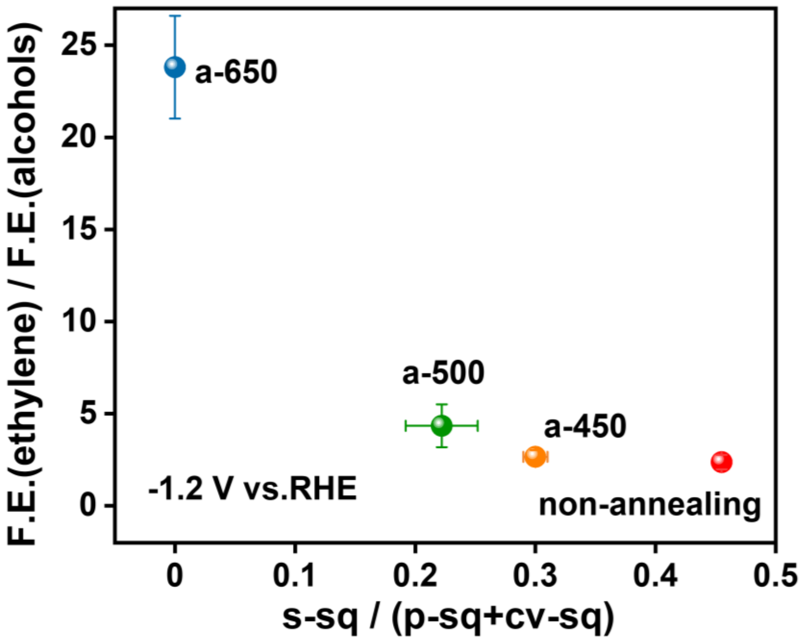

Supplementary Fig. 23 | Correlation between the active sites and activities. Ethylene/alcohols ratios as the function of the ratios of step-square sites to the sum of planar-square and convex-square sites at -1.2V vs. RHE. The error bars represent the standard deviation from three independent measurements.

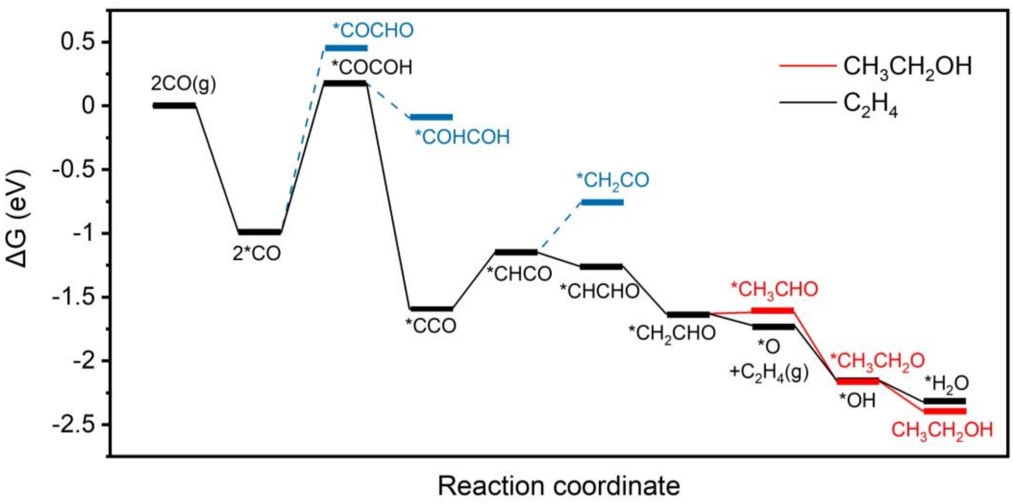

Supplementary Fig. 24 | Reaction Pathway. The reaction pathway for CO_2 electroreduction on p-sq.

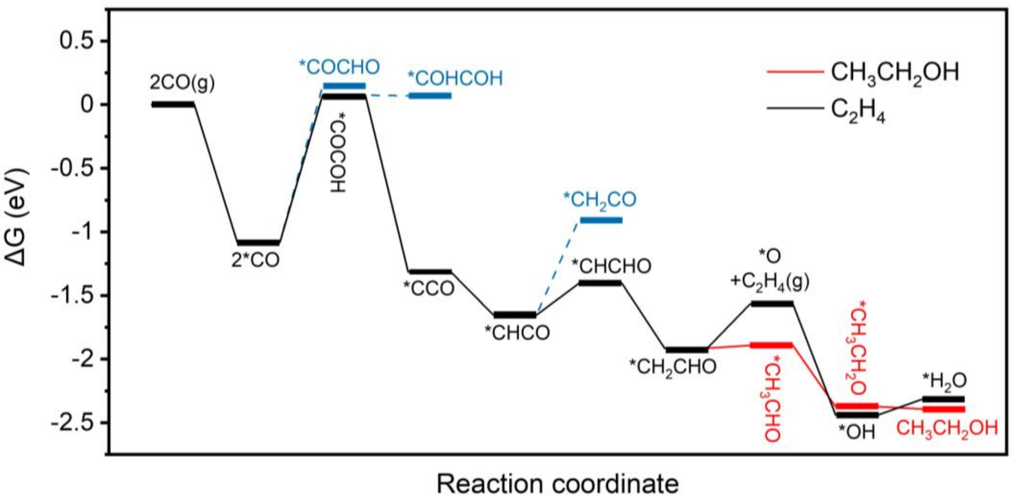

Supplementary Fig. 25 | Reaction Pathway. The reaction pathway for CO_2 electroreduction on s-sq.

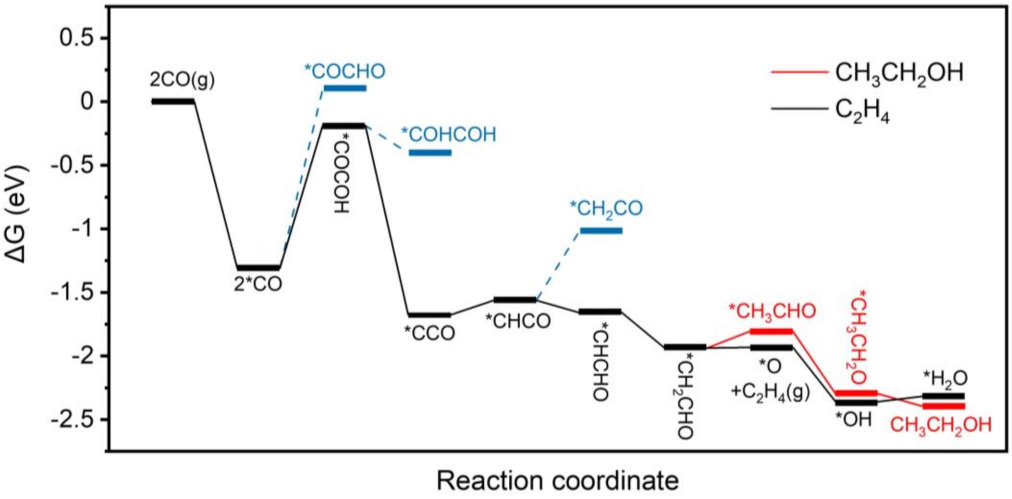

Supplementary Fig. 26 | Reaction Pathway. The reaction pathway for CO_2 electroreduction on cv-sq.

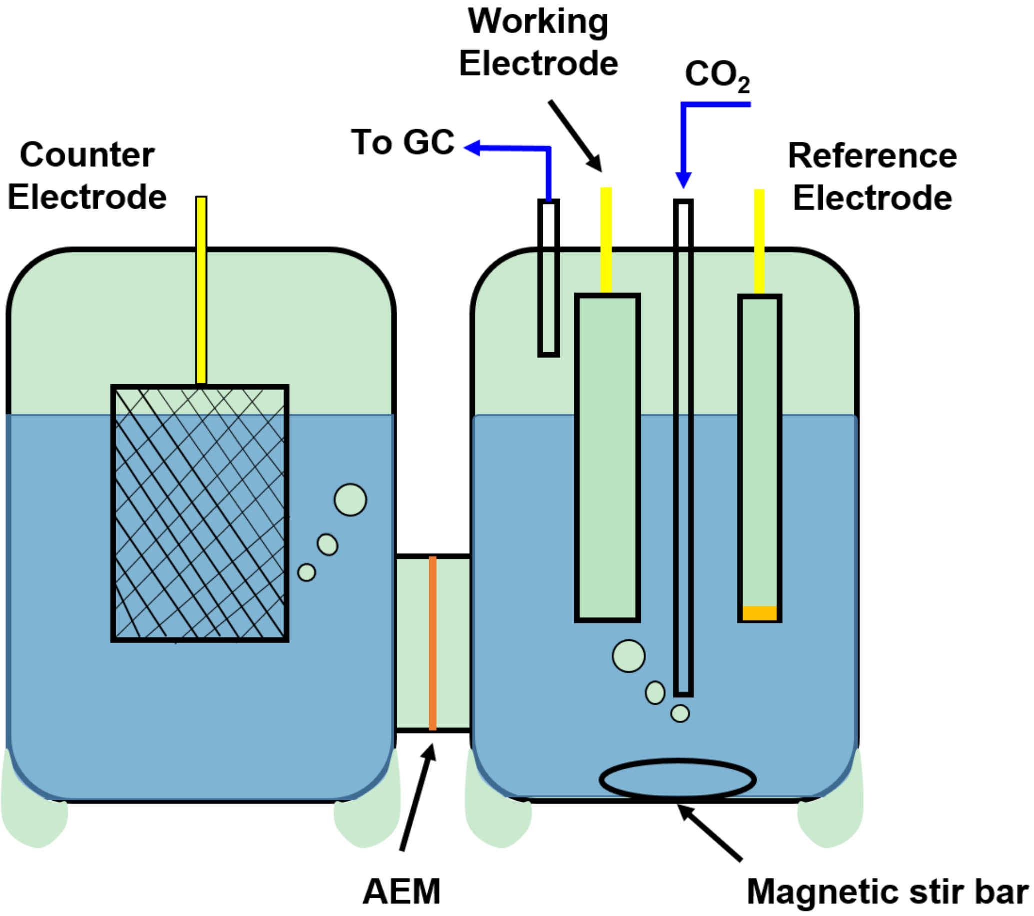

Supplementary Fig. 27 | Experimental setup. Schematic of the experimental setup used for CO_2 reduction.

Supplementary Table 6 | Reaction energies of CO-CO coupling in vacuum and with a layer charged water.

	Reaction energy in vacuum(eV)	Reaction energy with charged water(eV)
p-sq	1.02	-0.16
s-sq	1.27	0.16
cv-sq	1.10	0.11

Supplementary Table 7 | Reaction energies and reaction barriers of C-C coupling

Surface model	Reaction energy (eV)	Reaction barrier (eV)
(111)	0.68	0.85
(100)	-0.10	0.50
(221)	1.04	1.19
p-sq	-0.16	0.33
s-sq	0.17	0.72
cc-sq	0.11	1.11
cv-sq	0.09	0.38

Supplementary Table 8 | Gibbs free reaction energy on different surface models

Reaction free energy (eV)	p-sq	s-sq	cv-sq
$*C_2H_3O + H^+ + e^- \rightarrow *O + C_2H_4$	-0.09	0.35	0.01
$*O + H^+ + e^- \rightarrow *OH$	-0.43	-0.87	-0.43
$*OH + H^+ + e^- \rightarrow * + H_2O$	-0.16	0.12	0.04
$*C_2H_3O + H^+ + e^- \rightarrow *C_2H_4O$	0.01	0.02	0.13
$*C_2H_4O + H^+ + e^- \rightarrow *C_2H_5O$	-0.54	-0.48	-0.49
$*C_2H_5O + H^+ + e^- \rightarrow * + C_2H_5OH$	-0.23	-0.02	-0.09

Supplementary Table 9 | Ratios of active sites after annealing at different temperatures

	p-sq	s-sq	cv-sq
Non-annealing (%)	5.29	3.61	2.64
a-450K (%)	4.64	2.05	2.68
a-500K (%)	4.04	0.89	0.00
a-650K (%)	1.67	0.00	0.00

Supplementary Table 10 | Ratios of active sites after annealing at different temperatures

	p-sq				s-sq				cv-sq			
	0.1ns	0.2ns	0.3ns	ave	0.1ns	0.2ns	0.3ns	ave	0.1ns	0.2ns	0.3ns	ave
Non-annealing(%)	5.29				3.61				2.64			
a-450K (%)	4.01	4.72	5.19	4.64	2.13	1.89	2.13	2.05	2.60	2.83	2.60	2.68
a-500K (%)	3.15	4.61	4.36	4.04	0.73	1.21	0.73	0.89	0.00	0.00	0.00	0.00
a-650K (%)	1.13	1.88	2.00	1.67	0.00	0.00	0.00	0.00	0.00	0.00	0.00	0.00

Supplementary Table 11 | Cartesian Coordination of the planar-square structure

Unit cell:

	x /Å	y /Å	z /Å
x	18.40	0.00	0.00
y	0.00	7.64	0.00
z	0.00	0.00	18.25

Atom	x /Å	y /Å	z /Å
Cu	1.76	2.08	7.37
Cu	3.97	0.81	7.37
Cu	6.16	2.08	7.36
Cu	3.97	3.36	7.37
Cu	6.16	4.63	7.36
Cu	8.40	0.81	7.32
Cu	8.40	3.36	7.32
Cu	8.40	5.91	7.32
Cu	15.41	0.78	7.33
Cu	17.62	2.05	7.33
Cu	11.03	0.78	7.33
Cu	13.25	2.05	7.38
Cu	11.03	3.32	7.33
Cu	13.25	4.60	7.38
Cu	11.03	5.87	7.33
Cu	15.41	3.33	7.32
Cu	1.76	4.62	7.37
Cu	1.76	7.17	7.37
Cu	3.97	5.90	7.37
Cu	6.16	7.18	7.36
Cu	13.25	7.15	7.38
Cu	15.41	5.87	7.33
Cu	17.62	4.59	7.33
Cu	17.62	7.14	7.32
Cu	6.19	0.78	3.17
Cu	1.77	0.78	3.17
Cu	3.98	2.05	3.17
Cu	6.19	3.33	3.17
Cu	1.77	3.33	3.17
Cu	3.98	4.61	3.17
Cu	6.19	5.88	3.17
Cu	5.12	0.78	5.26

Cu	7.43	2.05	5.26
Cu	0.50	0.78	5.26
Cu	2.81	2.05	5.26
Cu	5.12	3.33	5.26
Cu	7.43	4.61	5.26
Cu	1.77	5.88	3.17
Cu	3.98	7.16	3.17
Cu	0.50	3.33	5.26
Cu	2.81	4.61	5.26
Cu	5.12	5.88	5.26
Cu	7.43	7.16	5.26
Cu	0.50	5.88	5.26
Cu	2.81	7.16	5.26
Cu	17.63	0.78	3.17
Cu	13.21	0.78	3.17
Cu	15.42	2.06	3.17
Cu	17.63	3.33	3.17
Cu	8.40	2.05	3.17
Cu	13.21	3.33	3.17
Cu	15.42	4.61	3.17
Cu	17.63	5.88	3.17
Cu	14.36	0.78	5.26
Cu	16.67	2.05	5.26
Cu	9.74	0.78	5.26
Cu	12.05	2.05	5.26
Cu	14.36	3.33	5.26
Cu	16.67	4.61	5.26
Cu	8.40	4.61	3.17
Cu	13.21	5.88	3.17
Cu	15.42	7.16	3.17
Cu	8.40	7.16	3.17
Cu	9.74	3.33	5.26
Cu	12.05	4.61	5.26
Cu	14.36	5.88	5.26
Cu	16.67	7.16	5.26
Cu	9.74	5.88	5.26
Cu	12.05	7.16	5.26
Cu	10.98	2.10	3.17
Cu	10.98	4.66	3.17
Cu	10.98	7.21	3.17

Supplementary Table 12 | Coordination (cartesian) of the step-square structure

Unit cell:

	x /Å	y /Å	z /Å
x	10.99	0.00	0.00
y	-1.83	7.55	0.00
z	0.00	0.00	25.22

Atom	x /Å	y /Å	z /Å
Cu	-0.10	2.41	5.49
Cu	2.34	3.00	8.16
Cu	-0.10	5.08	9.93
Cu	-0.71	4.93	5.49
Cu	1.73	5.52	8.16
Cu	0.51	2.56	9.93
Cu	1.73	4.19	5.93
Cu	0.51	1.23	7.71
Cu	2.95	1.82	10.38
Cu	0.51	3.89	12.16
Cu	2.34	1.67	5.93
Cu	-0.10	3.74	7.71
Cu	2.34	4.34	10.38
Cu	1.12	1.37	12.16
Cu	-1.32	7.45	5.49
Cu	1.12	8.04	8.16
Cu	-0.71	7.60	9.93
Cu	-0.71	6.26	7.71
Cu	1.73	6.85	10.38
Cu	1.12	6.71	5.93
Cu	-0.10	6.41	12.16
Cu	6.01	3.89	4.60
Cu	4.78	0.93	6.38
Cu	8.45	4.48	7.27
Cu	7.23	1.52	9.04
Cu	4.78	3.60	10.82
Cu	3.56	0.63	12.60
Cu	9.67	2.12	11.71
Cu	6.62	1.37	4.60
Cu	4.17	3.45	6.38
Cu	9.06	1.97	7.27
Cu	6.62	4.04	9.04
Cu	5.39	1.08	10.82

Cu	2.95	3.15	12.60
Cu	9.06	4.63	11.71
Cu	4.17	2.12	4.16
Cu	9.06	0.63	5.05
Cu	6.62	2.71	6.82
Cu	4.17	4.78	8.60
Cu	9.06	3.30	9.49
Cu	6.62	5.37	11.27
Cu	3.56	4.63	4.16
Cu	8.45	3.15	5.05
Cu	6.01	5.23	6.82
Cu	4.78	2.26	8.60
Cu	9.67	0.78	9.49
Cu	7.23	2.86	11.27
Cu	3.56	5.97	6.38
Cu	6.01	6.56	9.04
Cu	2.34	5.67	12.60
Cu	8.45	7.15	11.71
Cu	5.39	6.41	4.60
Cu	7.84	7.00	7.27
Cu	4.17	6.11	10.82
Cu	2.95	7.15	4.16
Cu	7.84	5.67	5.05
Cu	5.39	7.74	6.82
Cu	3.56	7.30	8.60
Cu	8.45	5.82	9.49
Cu	6.01	7.89	11.27

Supplementary Table 13 | Coordination (cartesian) of the concave-square structure

Unit cell:

	x /Å	y /Å	z /Å
x	10.40	0.00	0.00
y	0	7.73	0.00
z	0.00	0.00	27.25

Atom	x /Å	y /Å	z /Å
Cu	2.13	1.95	3.84
Cu	2.14	4.52	3.87
Cu	2.14	7.09	3.89
Cu	0.43	1.91	10.38
Cu	0.43	4.48	10.39
Cu	0.43	7.06	10.40
Cu	3.02	1.93	6.39
Cu	3.04	4.50	6.42
Cu	3.04	7.07	6.44
Cu	1.25	1.89	13.01
Cu	1.25	4.46	13.01
Cu	1.25	7.04	13.02
Cu	3.90	1.91	9.03
Cu	3.90	4.48	9.05
Cu	3.90	7.06	9.06
Cu	6.39	1.94	5.02
Cu	6.40	4.51	5.04
Cu	6.40	7.08	5.07
Cu	0.84	0.65	5.75
Cu	0.84	3.23	5.77
Cu	0.86	5.79	5.79
Cu	1.72	0.63	8.39
Cu	1.73	3.20	8.39
Cu	1.73	5.78	8.41
Cu	4.26	0.66	4.41
Cu	4.27	3.23	4.42
Cu	4.28	5.80	4.45
Cu	2.61	0.61	11.01
Cu	2.61	3.19	11.01
Cu	2.61	5.76	11.03
Cu	5.17	0.64	7.02

Cu	5.18	3.21	7.04
Cu	5.18	5.79	7.07
Cu	7.36	1.90	11.73
Cu	7.36	4.48	11.73
Cu	7.37	7.06	11.73
Cu	4.80	1.89	11.74
Cu	4.80	4.46	11.75
Cu	4.80	7.04	11.75
Cu	7.31	1.92	7.65
Cu	7.31	4.49	7.67
Cu	7.32	7.06	7.69
Cu	3.37	0.60	13.57
Cu	3.37	3.18	13.58
Cu	3.37	5.75	13.58
Cu	6.06	0.62	9.73
Cu	6.06	3.19	9.73
Cu	6.06	5.77	9.74
Cu	9.94	1.93	7.77
Cu	9.95	4.50	7.78
Cu	9.95	7.08	7.79
Cu	8.63	0.63	9.72
Cu	8.63	3.20	9.72
Cu	8.64	5.78	9.73
Cu	9.52	0.61	12.35
Cu	9.52	3.19	12.35
Cu	9.52	5.76	12.36
Cu	10.00	1.95	3.82
Cu	10.01	4.52	3.84
Cu	10.02	7.10	3.85
Cu	8.69	0.65	5.74
Cu	8.68	3.23	5.75
Cu	8.70	5.80	5.77

Supplementary Table 14 | Coordination (cartesian) of the convex-square structure

Unit cell:

	x /Å	y /Å	z /Å
x	10.20	0.00	0.00
y	0	7.71	0.00
z	0.00	0.00	27.00

Atom	x /Å	y /Å	z /Å
Cu	1.40	2.02	6.38
Cu	1.41	4.58	6.39
Cu	1.41	7.16	6.41
Cu	2.26	2.01	8.79
Cu	2.26	4.58	8.80
Cu	2.26	7.15	8.81
Cu	4.78	2.02	5.10
Cu	4.78	4.59	5.12
Cu	4.79	7.16	5.12
Cu	2.67	0.73	4.53
Cu	2.67	3.30	4.54
Cu	2.67	5.88	4.56
Cu	0.99	0.72	10.65
Cu	0.99	3.29	10.65
Cu	0.99	5.86	10.66
Cu	3.53	0.73	6.95
Cu	3.54	3.30	6.96
Cu	3.53	5.88	6.98
Cu	5.67	2.01	11.16
Cu	5.67	4.58	11.16
Cu	5.67	7.15	11.17
Cu	3.13	2.01	11.16
Cu	3.13	4.58	11.17
Cu	3.13	7.15	11.17
Cu	5.69	2.02	7.48
Cu	5.69	4.59	7.49
Cu	5.69	7.14	7.50
Cu	4.43	0.72	9.36
Cu	4.43	3.30	9.37
Cu	4.43	5.87	9.38
Cu	8.20	2.01	7.60

Cu	8.20	4.58	7.60
Cu	8.20	7.16	7.60
Cu	0.56	2.03	4.01
Cu	0.55	4.59	4.01
Cu	0.55	7.16	4.00
Cu	9.07	2.01	10.08
Cu	9.07	4.58	10.08
Cu	9.07	7.15	10.08
Cu	6.97	0.72	9.42
Cu	6.97	3.29	9.43
Cu	6.98	5.87	9.44
Cu	9.44	0.73	5.76
Cu	9.45	3.30	5.77
Cu	9.45	5.87	5.77
Cu	0.12	0.73	8.21
Cu	0.12	3.30	8.21
Cu	0.12	5.87	8.22
Cu	8.23	2.02	3.97
Cu	8.23	4.59	3.96
Cu	8.24	7.17	3.94
Cu	6.89	0.73	5.71
Cu	6.89	3.30	5.71
Cu	6.90	5.87	5.71
